# Cell Assembly in Self-foldable Multi-layered Soft Micro-rolls

**DOI:** 10.1038/s41598-017-17403-0

**Published:** 2017-12-22

**Authors:** Tetsuhiko F. Teshima, Hiroshi Nakashima, Yuko Ueno, Satoshi Sasaki, Calum S. Henderson, Shingo Tsukada

**Affiliations:** 1 NTT Basic Research Laboratories, NTT Corporation, 3-1 Morinosato-Wakamiya, Atsugi, Kanagawa, 243-0198 Japan; 2 Present Address: School of Chemistry, The University of Edinburgh, Scotland David Brewster Road, Edinburgh, EH9 3FJ United Kingdom

## Abstract

Multi-layered thin films with heterogeneous mechanical properties can be spontaneously transformed to realise various three-dimensional (3D) geometries. Here, we describe micro-patterned all-polymer films called micro-rolls that we use for encapsulating, manipulating, and observing adherent cells *in vitro*. The micro-rolls are formed of twin-layered films consisting of two polymers with different levels of mechanical stiffness; therefore they can be fabricated by using the strain engineering and a self-folding rolling process. By controlling the strain of the films geometrically, we can achieve 3D tubular architectures with controllable diameters. Integration with a batch release of sacrificial hydrogel layers provides a high yield and the biocompatibility of the micro-rolls with any length in the release process without cytotoxicity. Thus, the multiple cells can be wrapped in individual micro-rolls and artificially reconstructed into hollow or fibre-shaped cellular 3D constructs that possess the intrinsic morphologies and functions of living tissues. This system can potentially provide 3D bio-interfaces such as those needed for reconstruction and assembly of functional tissues and implantable tissue grafts.

## Introduction

Three-dimensional (3D) biocompatible architectures are of interest in terms of providing an interface with cells for single-cell assay^1–3^, tissue engineering^4,5^, and transplantation therapeutics^1,6^. They have proven beneficial for providing extracellular micro-environments that mimic hierarchical structures with spatially confined physiological environments of human tissue such as neural tissues^4^, muscle fibres^4,5^, and blood vessels^7,8^, In particular, polymeric structures are expected to create *ex vivo* 3D cellular constructs with cell-to-cell connections, intrinsic morphologies, and functions, thanks to their high elasticity, biocompatibility, and transparency^9,10^. Although various types of 3D fabrication processes including stereo-lithography and laser micromachining techniques have been proposed, there is still a technical limitation as regards constructing micro- or nano-scale polymer structures with controlled 3D geometries.

To achieve 3D bottom-up fabrication, the mechanism of bimetal film transformation^2,3,11^ has been utilized for polymer-based self-assembly with highly precise geometry^12^. The simple curve of polymeric bilayer films with heterogeneous mechanical properties enables assembly into various 3D forms such as pyramidal^13^, tubular^14–17^, helical^18–20^, and plant-inspired complex structures^21–24^. However, it is technically difficult to apply these 3D geometries to an interface with cells owing to cytotoxic release processes with chemical^15–18,22^, thermal^13,14,19–21,24^ or electrical^23^ triggers, which limit the building of 3D cell-laden architectures. Thus, an alternative biocompatible release method is needed if we are to undertake investigations utilizing manual rolling^25^, swelling detachment^26,27^, intrinsic cell traction force^28^, and the release of a stretched elastomeric substrate^29–31^. Both cell-friendly polymers and biocompatible batch release will surely be used to create *ex vivo* 3D cellular architectures greater than a centimetre in size with cell-cell connections, intrinsic morphologies, and various functions^9^.

In this study, we show that multi-layered polymeric films with heterogeneous mechanical properties can form self-folded rolled shapes (micro-rolls) by integrating them with the biocompatible batch release of a hydrogel-based sacrificial layer^3,32^. We used alginate hydrogel as the sacrificial layer for the micro-patterned films, because it can be dissolved by adding chelating agents, thus achieving a spontaneous and non-cytotoxic batch release process with arrayed cell-laden films^33^. The films with sacrificial layers consist entirely of transparent and biocompatible polymers, namely silk fibroin hydrogel, poly(chloro-p-xylylene) (parylene-C), and calcium alginate (Ca-alginate). We selected silk fibroin crystalline polymer reconstituted from *Bombyx mori* due to its mechanically robust features^34^, high optical transparency^35^, and excellent FDA/USP-approved biocompatibility as regards implantation^36,37^. Parylene-C is also an FDA-approved, chemically inert, and nonbiodegradable crystalline polymer, which is extensively used for conformal coatings for medical implants and for mobile interfaces with cells^33,38^ and tissues^39,40^ with low cytotoxicity. While they are not deformable themselves, the combination of silk fibroin with conformally deposited parylene-C will function as a mechanically heterogeneous bilayer that can transform micro-patterned thin film into controlled 3D geometries. After releasing the sacrificial layer in a non-cytotoxic process, the micro-patterned films are autonomously self-folded into cylindrical shapes based on differential strain gradients which depend on the film thickness. Various 3D cell-laden microstructures formed from two-dimensional (2D) geometrical micro-patterns enable the cells to migrate, connect with each other to form the desired 3D architectures, and synchronize their behaviour within a micro-cavity. We also use various cell-lines and primary cultured cells to demonstrate that they can reconstitute the intrinsic cellular morphologies and functions. These results will potentially lead to an effective and versatile way of realising 3D bio-interfaces for such applications as the reconstruction of functional tissues and implantable tissue grafts.

## Results

**Figure 1:**
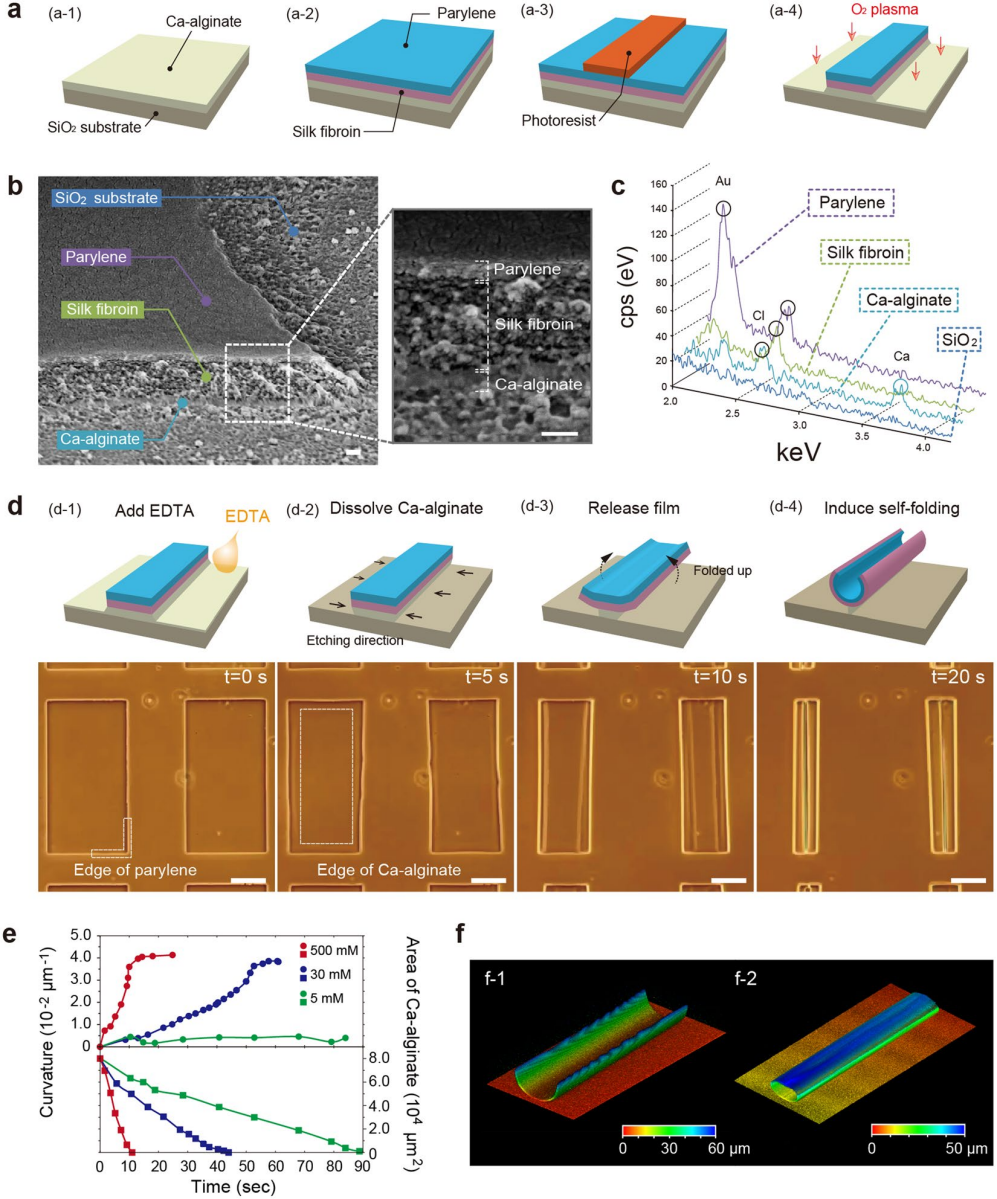
Versatile self-foldable film used to form cylindrical shapes called “micro-rolls.” (**a**) Schematic illustration showing the process for fabricating multi-layered thin film. A Ca-alginate layer was spin-coated on a SiO_2_ substrate (a-1). Silk fibroin was spin-coated and gelated. Ca-alginate and silk fibroin layers were coated with parylene-C by CVD (a-2). After the photoresist had been micro-patterned (a-3), the micro-pattern of the multi-layered film was fabricated with O_2_ plasma etching (a-4). (**b**) Low-magnification SEM images of micro-patterned films on a SiO_2_ substrate. The white box indicates enlarged areas. (**c**) Corresponding EDX spectra over the same area selected in (**b**). (**d**) Explanatory illustration (top) and phase-contrast images (bottom) of sequential self-folded micro-rolls. Removal of the sacrificial Ca-alginate layer triggered the spontaneous self-folding of the micro-rolls. (**e**) Time-dependent plot of curvature value of micro-rolls (1/*ρ*) and remaining area of Ca-alginate underneath self-folded 200 × 400 μm^2^ films versus various final concentrations of EDTA solution. (**f**) Confocal images of micro-rolls with different curvature radii: (f-1) 171.7 ± 3.4 μm, (f-2) 25.0 ± 1.79 μm (mean ± s.d., sixteen different micro-rolls). Scale bars: 400 nm in (b-1), 100 nm in (b-2), and 100 μm in (**d**).

We fabricated self-foldable films consisting of several layers of mechanically stable and biocompatible polymers, namely parylene-C and silk fibroin^35^, on an underlying sacrificial layer of Ca-alginate^4,33^. As the fabrication method (Fig. 1a, Supplementary Fig. S1), Ca-alginate and silk fibroin were spin-coated on a SiO_2_ substrate and gelated using both methanol treatment and water-annealing processes in accordance with previously reported protocols^41–43^. Then, the tops of the films were laminated with parylene-C layer by chemical vapour deposition (CVD). Thereafter, the multi-layered film was micro-patterned photolithographically, and etched with oxygen plasma through a photoresist mask, resulting in highly defined geometries^28,33,41,44^. SEM images showed clearly identifiable trilaminar films (Fig. 1b, Supplementary Fig. S2a). Energy dispersive X-ray (EDX) spectra showed that the Ca-alginate layer possessed Ca peaks around 3.7 keV and that the parylene-C layer possessed a Cl peak at 2.6 keV (Fig. 1c). The silk fibroin middle layer also exhibited a peak originating from Cl, implying that the deposited parylene-C penetrated the silk fibroin layer and enhanced the mutual attraction force between the two films.

To realise self-folding 3D architectures, we dissolved the Ca-alginate sacrificial layer by adding ethylene-diaminetetraacetic acid (EDTA). The Ca-alginate layer gradually shrank from the edges of the films, releasing the remaining bilayers and initiating the formation of tubular structures through self-folding (Fig. 1d, Supplementary Fig. S3, Supplementary Movie SM1). The time dependence of the dissolving area of the Ca-alginate layer indicated that the self-folding speed was sensitive to the ambient concentration of EDTA (Fig. 1e)^45^. The curvature of the self-folded micro-rolls (1/*ρ*) reached an equilibrium value of approximately 0.04, which was delayed by more than 10 sec after the completion of the Ca-alginate dissolution. Since the self-folding process was driven by the difference in stiffness of the bilayer components in the completely swollen state, the slow dissolution of Ca-alginate resulted in the failure of the film transformation. In addition to the slow dissolution of the Ca-alginate, the films with non-gelated silk fibroin layers did not become cylindrical but remained flat. Confocal reconstructed images showed that the dimensions of the micropatterns had a noticeable effect on the diameter of the resulting tubular structures (Fig. 1f).

**Figure 2:**
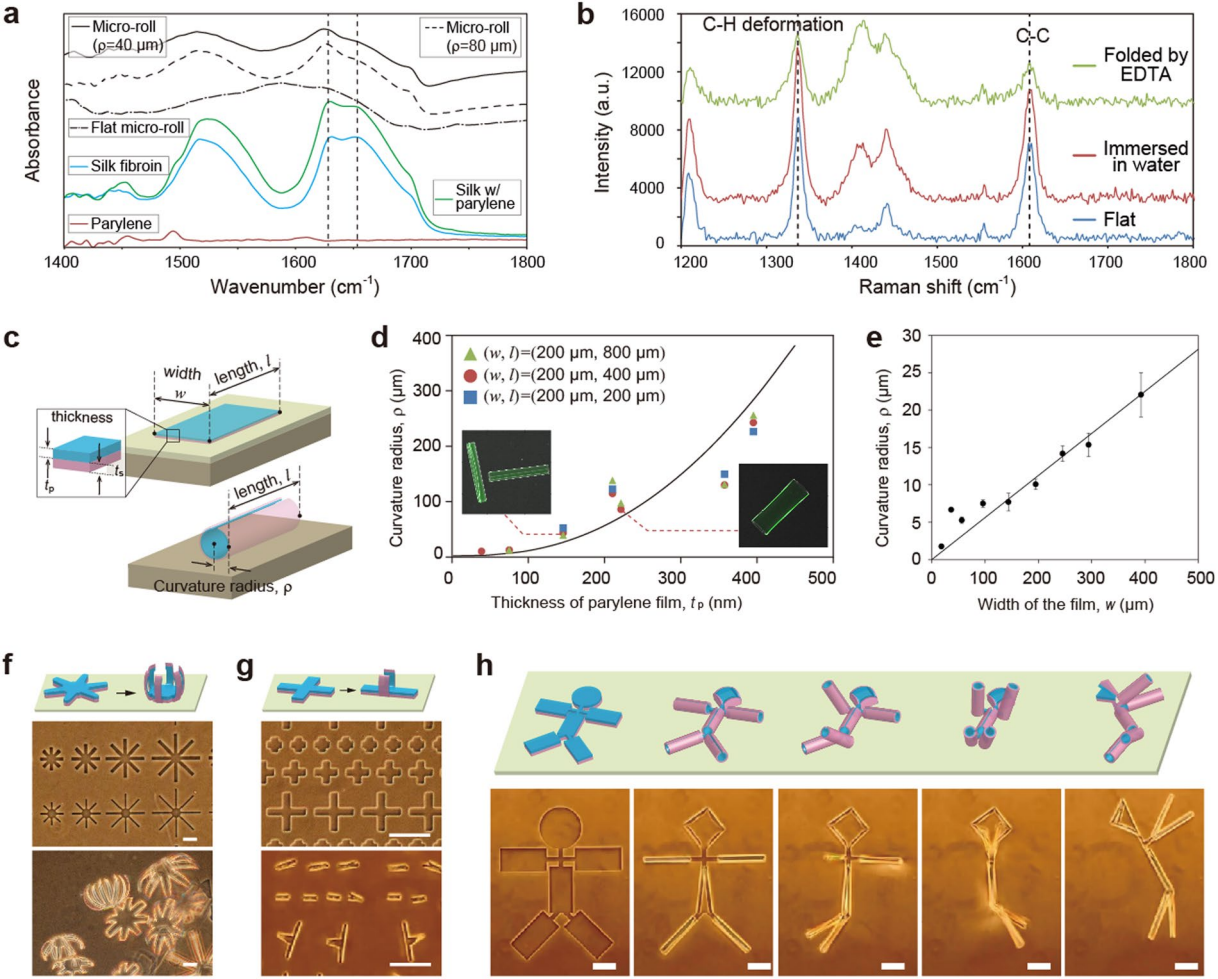
Characterisation of self-folded micro-rolls. (**a**) FTIR spectra of crystalline silk fibroin in micro-rolls. Resonance absorbances centred at 1,641 and 1,621 cm^−1^ indicate a random coil conformation in amorphous silk protein and the helical β-sheet conformation of annealed protein, respectively. (**b**) Raman spectroscopy performed to check the characteristics of parylene-C. Peaks near 1,330 and 1,610 cm^−1^, respectively, are characteristic of C-H and C-C deformation attributed to parylene-C. (**c**) Schematic illustration of length, width, thickness, and curvature radius of micro-rolls. (**d**) Plot of average curvature radius of free-floating, self-folded 200 × 200, 200 × 400, and 200 × 800 μm^2^ films versus various parylene thickness (*t*_p_). The standard deviation (s.d.) values of more than twelve different micro-rolls were less than 2.64. A theoretical curve derived from Eq. (1) is overlaid on the plots. (**e**) Plot of average radius of curvature of self-folded films with a constant aspect ratio (*w*/*l* = 0.5) versus shorter length (*w*) value. A linear approximate line with a zero *Y*-intercept is overlaid. The results are shown as the mean ± s.d. values of more than thirteen different micro-rolls. (**f**–**h**) Schematic images and experimental optical images of the versatility of self-assembly for higher-order 3D macroscopic structures. 3D assembly of micropatterned films, for example flower (**f**), cross (**g**), and doll-shapes (**h**), are produced by the single step of dissolving Ca-alginate. Scale bars represent 100 μm.

We characterized the molecular conformation of the self-folded micro-rolls using FTIR and Raman spectroscopy. Silk fibroin is widely used as a temporary and soluble supporting thin film because it is amenable to aqueous processing such as methanol treatment^42^ or water-annealing process^43^. It is also programmable with dissolution rates, depending on the inducement of β-sheet conformation in crystalline silk. The FTIR spectra from 1450 to 1700 cm^−1^ are assigned to the absorption of the peptide backbones in silk fibroin, which was methanol-treated and water-annealed (Fig. 2a). All the spectra of the films containing silk fibroin possessed an absorbance centred at 1625 cm^−1^, which is characteristic of the antiparallel β-sheet conformation in crystalline silk. However, the folded micro-rolls exhibited a reduction in absorbance around 1650 cm^−1^, suggesting a transition from the helical conformation of amorphous silk to a β-sheet-rich conformation^35^. We did not observe any specific differences in the wavelengths of the peaks of the micro-rolls with the curvature radius of 40 μm and 80 μm. In addition, thanks to its high elastic modulus the β-sheet-rich matrix of the silk fibroin was maintained without a phase transition after the self-folding step. On the other hand, when the silk fibroin layer was not treated, the films with fewer peaks around 1650 cm^−1^ became cylindrical, and returned to their original flat state after incubation overnight. The flat micro-rolls did not possess the absorbance peaks centred at both 1625 and 1650 cm^−1^. This is partly because the silk fibroin that did not undergo chemical, photo-initiated, or shear-stress mechanical crosslinking reactions experienced no polymorphic transition to induce β-sheets, resulting in higher water solubility^34,35,42^. Accordingly, treatment with methanol and water annealing to induce a β-sheet-rich conformation is an indispensable process as regards maintaining the shape of the micro-rolls and making them sufficiently durable for long-term incubation. In contrast, the Raman spectra included peaks at 1336 and 1608 cm^−1^, corresponding to the deformation of the C-H and C-C bonds in parylene (Fig. 2b)^46^. These peaks were present in flat film under both dry and wet conditions and were observed even in folded states as shown in Supplementary Fig. S2b, thus implying that the parylene layer displayed no defects. Consequently, these results indicate that the invariant chemical components of both parylene-C and gelated silk fibroin hydrogel ensure the structural stability of micro-rolls after the dissolution of the Ca-alginate sacrificial layer.

We applied quantitative predictions of 2D designs to the final geometry to allow us to control the micro-roll architecture. Since the underlying mechanism of self-folding is the stiffness mismatch in the bilayer, the curvature radius of the micro-rolls (*ρ*) was modelled using Timoshenko’s bimorph beam theory^47^. Whereas Flory–Huggins’s thermodynamic framework is applied to the thermoresponsive or swelling behaviour of homogeneous films with stiffness gradients^16,26,48^, this theory based on the bending moment of thin bilayer film beams is employed to predict the curvature radius based on the elastic modulus, layer thickness (*t*), and strain (*ε*) as follows^19,21^:

1 $$\rho =\frac{d[3{(1+m)}^{2}+(1+mn)\{{m}^{2}+{(mn)}^{-1}\}]}{6({\varepsilon }_{{\rm{p}}}-{\varepsilon }_{{\rm{s}}}){(1+m)}^{2}}$$

where *n* is the relative elastic modulus, *m* is the ratio of the bilayer thickness (*m* = *t*_p_/*t*_s_), *d* is the total thickness (*d* = *t*_p_ + *t*_s_), and the subscripts “p” and “s” denote parylene and silk fibroin, respectively (Fig. 2c, Supplementary Information 2–3). For simplicity, *t*_s_ was kept constant and *ρ* was measured in the equilibrium state as a function of varying parylene film thickness (*t*_p_). The experimental *ρ* value is plotted as a function of *t*_p_ ranging from 40 to 500 nm (Fig. 2d, the standard deviation is less than 2.64 μm). We measured the elastic modulus of silk fibroin in the completely swollen state, which is a key parameter for simulating the final 3D geometry of micro-rolls. The value of the elastic modulus calculated from tensile stress versus engineering strain curves by using a tension testing device equipped with 50 N load cells was estimated to be approximately 80 MPa (Supplementary Fig. S4e). Additionally, *ρ* is proportional to the width (*w*) when the aspect ratio is constant (*w*/*l* = 0.5), and constant with variable length (*l*) when both *w* and *t* are constant (Fig. 2e, Supplementary Fig. S4f,g). Since the experimental values of *ρ* mostly follow the trend predicted by equation (1), we concluded that the curvature radii of micro-rolls can be analytically predicted using Timoshenko’s theory.

In addition to the cylindrical shape, various 3D geometries can be controlled and self-folded depending on the design of the 2D micropatterns. For instance, flower and cross micropatterns were transformed into grippers and T-junctions, respectively (Fig. 2f,g). By combining rectangles with cross-shaped hinges, a unique doll-shaped 3D structure was self-folded where the rectangles correspond to the body and limbs, and a circle forms the head (Fig. 2h). In addition, the cross portion connecting the arms retained the backbone shape, consequently making it possible to maintain the 3D doll geometry. Hence, by incorporating the branched structure in the pattern interior, it is possible to produce more complex 3D shapes such as doll-shaped, zigzag-shaped, and caged skeletal frames (Supplementary Fig. S5, Supplementary Movie SM2).

**Figure 3:**
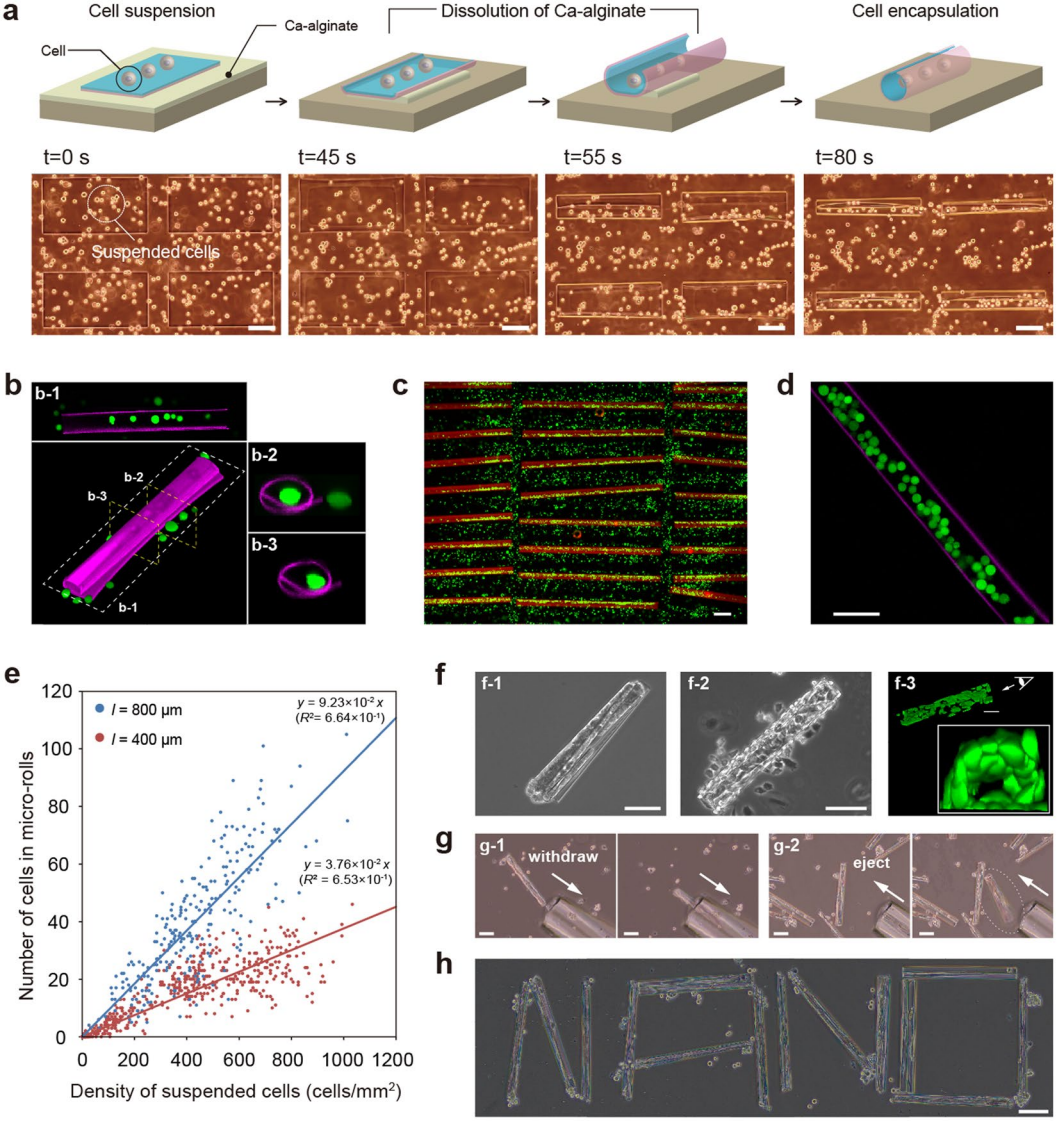
Encapsulation and incubation of multiple mammalian cells inside micro-rolls. (**a**) Time-lapse schematic and optical images illustrating the cell encapsulation. Once EDTA solution is added, the films transform and encapsulate the cells suspended on the film surface. (**b**) Confocal reconstructed and cross-sectional (b-1, b-2, b-3) images of cell-laden micro-rolls where *ρ* is approximately 20 μm. The silk fibroin layers in the micro-rolls and encapsulated cells were labelled with Qdot 655 and calcein, respectively. (**c**) A batch release of films produced an array of cell-containing micro-rolls. (**d**) Confocal cross-sectional images of cell-laden micro-rolls where *ρ* is approximately 40 μm. (**e**) The number of encapsulated cells inside micro-rolls with *l* = 400 μm (red dots), 800 μm (blue dots) with respect to the cell density of suspended cells. (**f**) Phase-contrast and reconstructed confocal 3D images of cells inside micro-rolls with *ρ* = 40 μm after 24 h incubation. HEK cells in micro-rolls formed cell fibres (f-1). By contrast, CHO cells in micro-rolls formed hollow structures (f-2, f-3). The enlarged confocal image on the right shows a cross-sectional view of a hollow structure. (**g**) Handling 400 μm-long cell-laden micro-rolls in culture medium. The micro-rolls can be withdrawn, relocated, and ejected with a picolitre flow using glass capillaries. (**h**) Micrograph of assembled cell-laden micro-rolls in culture medium. Scale bars: 100 μm in (**a**–**g**), 1 mm in (**f**).

The key challenge when fabricating cell-laden micro-rolls is to realise tubular structures with cyto-compatibility and to confirm the cell function within the micro-rolls. For a microscopic evaluation of biocompatibility and an investigation of the morphological and migration properties, we encapsulated two types of cell-lines within the micro-rolls: Chinese hamster ovary (CHO) and human embryonic kidney (HEK) cells. To produce micro-rolls with a curvature radius of approximately 40 μm, we employed parylene and silk fibroin with thickness of 145 and 210 nm, respectively. With the addition of EDTA, the cells on the 2D film surface were encapsulated inside the 3D micro-rolls through the self-folding of the bilayer (Fig. 3a, Supplementary Fig. S6, Supplementary Movie SM3). Confocal cross-sectional images revealed that arrayed micro-rolls encapsulated a large number of cells at a time and separated them from cells outside the patterned area (Fig. 3b–d, Supplementary Movie SM4). A live/dead assay revealed that more than 95% of the cells were alive inside the micro-rolls, indicating that the self-folding and encapsulation process is sufficiently gentle to avoid cell damage. Therefore, arrayed micro-rolls have the potential to create highly parallel assays for investigating cellular behaviours including proliferation and differentiation. Since the curvature radius and length of the micro-rolls are controllable, the number of encapsulated cells is easily adjusted by varying the suspended cell density and the micro-roll volume (Fig. 3e).

The high biocompatibility and transparency of the micro-rolls enabled encapsulated cells to be stably incubated inside the rolls and allowed the observation of cell behaviour such as migration and proliferation, using various types of microscopy. During the incubation, the encapsulated cells migrated and proliferated only within the micro-rolls and there was no migration out. This allows the formation of cell-cell contacts along the inner surfaces of the micro-rolls. The micro-rolls with encapsulated cells provide not only the transparency needed for microscope observations, but also sufficient mechanical strength to withstand the cell traction force. This phenomenon is comparable to that found in previous studies on not only cell encapsulation^2,3,49^, but also migration^50^ and proliferation^51^ within strain-engineered inorganic nano-membranes that offer a 3D environment for neural cells with a well-defined geometry. HEK cells exhibit relatively strong cell-to-cell contact, and so the cells in a micro-roll aggregated and completely filled the micro-roll cavity after 1 day of culture (Fig. 3f- 1). In contrast, CHO cells are likely to exist individually, meaning that they adhered preferentially to the inner surfaces of the rolls and formed hollow vessel-like constructs (Fig. 3f- 2, f-3). The integration with various 3D geometries of cell-aggregates shown in Supplementary Fig. S6 and encapsulated endothelial cells has potentials to produce architectures that mimic micro-scale vasculatures^52^. Moreover, the shapes of the cell clusters differed depending on the micro-roll volume and the cell-line’s adhesive properties. As firstly exploited in Si/SiO_2_ micro-rolls in a study of yeast cell behaviour^53^, the high transparency of the micro-rolls’ materials did not interfere with the light paths of microscopes. Therefore, it is possible to equip both upright and inverted microscopes with a micro-roll system for the long-term time-lapsed observation of encapsulated cells.

A picolitre fluid flow with a glass capillary enabled the manipulation and relocation of cell-laden micro-rolls by smoothly withdrawing the structure into the capillary and releasing it at the target location (Fig. 3g, Supplementary Fig. S7, Supplementary Movie SM5). While being conveyed inside the glass capillaries, the micro-rolls stably maintained their cylindrical shapes and held the cells without them being buckled or bent. Therefore, individual cells inside the micro-rolls could be collected, placed like building blocks, and successfully transported to the target position to induce cell-to-cell contact (Fig. 3h). The cell behaviour after long-term incubation is totally dependent on the surface modification of the substrates to which the micro-rolls were conveyed (Supplementary Fig. S6b–d). The HEK cells located on the ECM-coated substrates proliferated and migrated outside the micro-rolls. In contrast, the cells transferred to MPC polymer-coated substrates completely filled the micro-roll cavity, and then migrated onto the outer silk fibroin surface of the micro-rolls. Note that the cells on micro-rolls were manipulated without stripping them from their growth surfaces, which allowed the adhesive state of the manipulated cells to be preserved. This has the potential to facilitate high-order cellular assembly for potential use in tissue engineering by enabling the manipulation of individual cell types into any desired multi-cell-line combination.

**Figure 4:**
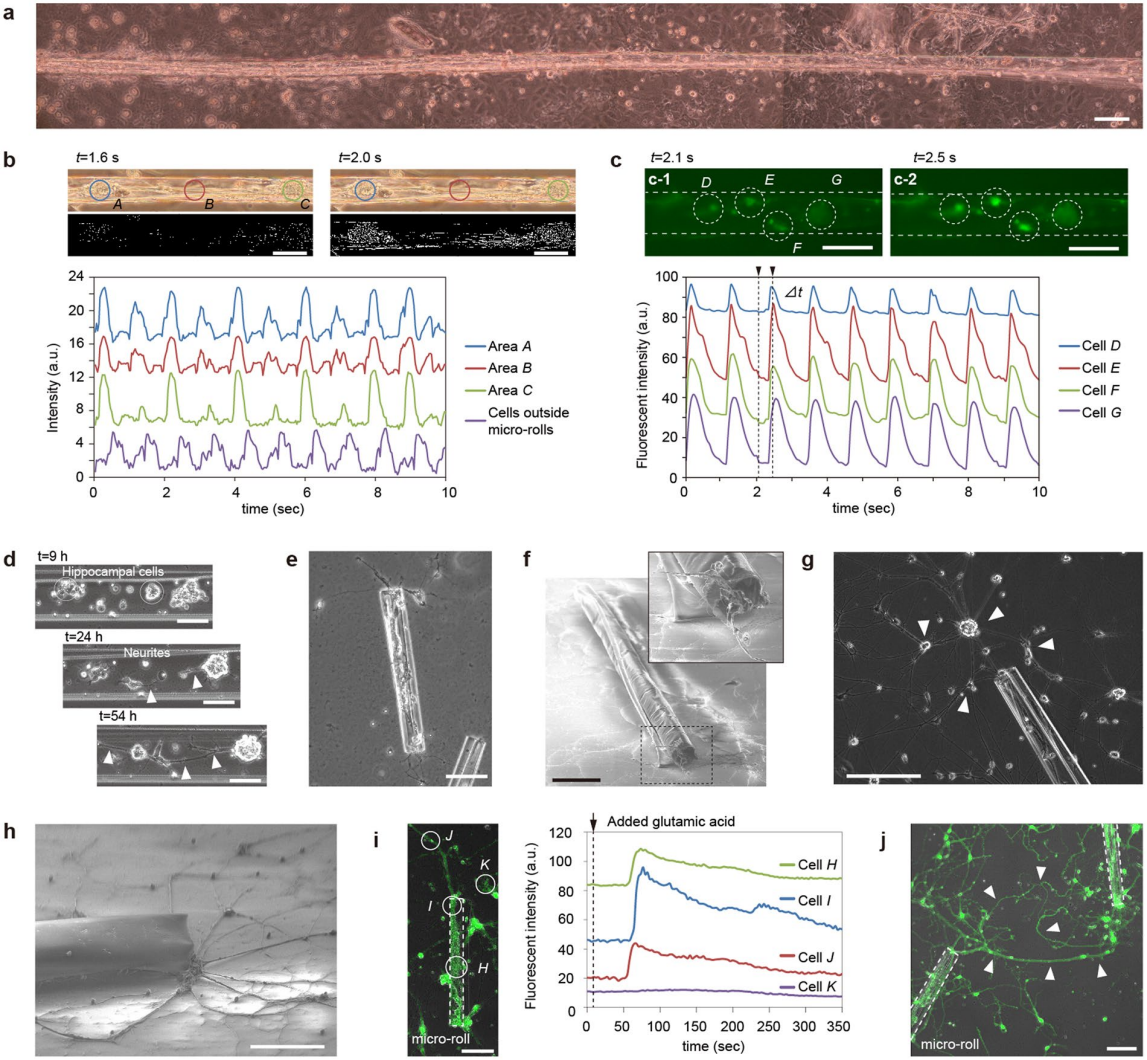
Intrinsic cellular morphologies and functions of primary cells in micro-rolls. (**a**) Optical image of more than 20 mm-long primary rat cardiomyocytes contained in micro-rolls. Primary rat cardiomyocytes gathered and formed into a tubular structure along the longer side of a micro-roll after 5 days of culture. Seven images taken independently are synthesized in a line. (**b**) Time-dependent displacement intensity profiles of the spontaneous contraction of the myocytes in the segment shown in the above bright-field images. (**c**) Relative fluorescence intensity profiles of Ca^2+^ oscillations in cardiomyocytes labelled with Fluo-4. (**d**) Time-lapsed images of hippocampal cells encapsulated in micro-rolls. Note that the presence of somas is highlighted by white circles, and the subsequent formation of extended neurites within the micro-rolls is indicated by a white arrowhead. (**e**,**f**) Phase-contrast (**e**) and SEM (**f**) images of hippocampal cell-laden micro-rolls cultured on MPC-polymer-coated dishes. The enlarged figures in the top right in (**f**) shows the guidance of neurites outside a micro-roll. (**g**,**h**) Phase-contrast (**g**) and SEM (**h**) images of hippocampal cell-laden micro-rolls cultured on PDL/laminin-coated dishes. The arrows indicate the external neurons connected to the neurites protruding from the micro-rolls. (**i**) Relative fluorescence intensity profiles of Ca^2+^ oscillations in hippocampal cells labelled with Fluo-4 and stimulated by adding glutamic acid in the segment shown in the confocal fluorescent image. (**j**) Merged micrograph of confocal and phase-contrast images of hippocampal cell-laden micro-rolls that were manipulated and assembled using glass capillaries. The arrows indicate the extended neurites labelled with calcein-AM bridged between neighbouring micro-rolls. Scale bars: 100 μm in (**a**–**g**,**i**,**j**), and 50 μm in (**h**).

We investigated physiological functions using two primary rat cells: cardiomyocytes and neural cells. In contrast to the cell lines, neither primary hippocampal cells nor cardiomyocytes proliferate spontaneously after incubation, and their cell bodies took their position only in the micro-rolls. Primary cardiomyocytes agglomerated and formed a tubular structure in 20 mm-long micro-rolls after 5 days of culture (Fig. 4a). Figure 4b shows optical images and time-dependent displacement (black-and-white images) at corresponding pixel locations, which we estimated by subtracting the average intensity from all the images in a stack (Supplementary Fig. S8, Supplementary Movie SM6). We observed that the intensity corresponding to the displacement revealed that all the cardiomyocytes connected inside the micro-rolls synchronously contracted in the same period at approximately 1 Hz. A time-lapse fluorescence image measurement of fluo-4-labelled cells indicated that the oscillation of intracellular Ca^2+^ propagated and was synchronized within the micro-rolls (Fig. 4c). The microenvironment provided by micro-rolls of any length could regulate the cell migration and cell-cell interactions and so drive intrinsic morphologies and functions.

Primary hippocampal cells were also encapsulated to reconstitute neural tissues. First, the encapsulated cells aggregated to form spheroids, and subsequently extended their dendrites in the micro-roll direction (Fig. 4d, Supplementary Movie SM7). After 3 days of culture, the neurite-mediated cell-cell connection between the spheroids resulted in the formation of unidirectionally oriented neural cell fibres (Fig. 4e). Somas and their neurites were both located inside the micro-rolls without any protrusion (Fig. 4f). Interestingly, the neurites were further extended outside the micro-rolls and connected with neighbouring cells (Fig. 4g,h). We used immunostaining and fluorescence microscopy to study the cell morphology with neurite outgrowth and intracellular components. Actin fibres were probed with Alexa Fluor 594-conjugated phalloidin (Supplementary Fig. S9a,b, right). Somatic, dendritic, and axonal proteins distributed across the cytoskeletons were visualised with Alexa Fluor 488-conjugated Pan Neuronal Marker monoclonal antibody (Supplementary Fig. S9a,b, centre). They demonstrated that both somas and their neurites were positionally restricted within the micro-rolls, and adhered around the circumference of the micro-rolls. As shown in Supplementary movie SM7, the encapsulated primary hippocampal cells firstly aggregated to form spheroids. Since the location of spheroids of hippocampal cells was random, some spheroids were located in the edge of micro-rolls (S9a, centre), and the others were evenly distributed inside the micro-rolls (S9b, centre). Regardless of the location of spheroids, their dendrites were extended and evenly distributed in the micro-roll after long-term incubation (S9a-b, right). We also evaluated the textile orientation of the neurite and actin fibre of evenly distributed hippocampal cells and their neurites within the micro-rolls quantitatively. We undertook an image analysis of projected images using a custom fast Fourier transform (FFT) with a Gaussian filter (Supplementary Fig. S9c,d). While the brightness distribution of the FFT image of the neurites of the hippocampal cells in the bulk was isotropic, that of the inner neurites peaked in the long axis direction of the micro-rolls. We also found that textiles fabricated using actin fibres were highly orientated towards the long axis of the micro-rolls. In contrast, when the cells were grown on a flat substrate, the arrangement and location of somas and neurite outgrowths appeared to be random. These results quantitatively suggest that the micro-rolls provide the geometric confinement of dense cell aggregates and the unidirectional alignment of neurites.

To further investigate the accessibility of neurites to the outer surface, we examined cellular responses to external stimulation with added glutamic acid. When the glutamic acid receptors in the hippocampal cells were activated, intracellular Ca^2+^ ions were detectable by calcium indicators using confocal laser scanning microscopy. Fluo-4 labelled cells exhibited spontaneous Ca^2+^ oscillations that were synchronized across the cells via synaptic connections, whereas the cells that were not connected via neurites showed no oscillation signals (Fig. 4i). This result indicates that the encapsulated cells come into direct contact and build up a cellular network with the cells outside of the micro-rolls through hollow structures in order to transmit and exchange information with their neighbouring cells. After 2 weeks of culture, the extended neurites from the manipulated cell-laden micro-rolls were connected and bridged to realise cell-to-cell contact between neighbouring micro-rolls (Fig. 4j). This reconstitution of a neurite-mediated network is also reasonably consistent with and equivalent to previous reports on Al_2_O_3_/SiO/SiO_2_^54^, Si/Ge^55^, or GaAs/InGaAs^56^ nanomembrane microtube structures with guided encapsulated neurons and their elongated neurites. With daily medium exchanges and cell maintenance, the biocompatibility of silk fibroin and parylene ensured the long-term culture (>1 month) of encapsulated cells without cytotoxicity. Consequently, the relocated cells can recreate intercellular communication with the cells outside of micro-rolls, serving as building units of higher-order tissue assemblies and potential implantable grafts *in vivo*.

## Discussion

In summary, we demonstrated that a multi-layered polymer film could change its shape into a programmed 3D configuration dependent upon its original 2D geometry. The self-folding mechanism is based on the bending of bilayer films and is potentially applicable to any constituent materials. Moreover, by combining this characteristic with other designs that undergo significant dimensional changes, it would be possible to construct more complex 3D artificial tissues. Notably, owing to the higher elasticity of parylene and silk fibroin, the micro-rolls help to develop a fibre-shaped cellular construct and as a result the contractile motion of the primary myocytes and synaptic connections of highly oriented primary neural cells could be achieved. This method could be expanded to many other adherent cell lines for the reconstruction of fibre-shaped functional tissues that mimic muscle fibres^57^, blood vessels^58,59^, and nerve networks *in vitro* ^60,61^.

All the polymeric materials we used for our micro-rolls, including the sacrificial layer, are transparent, biocompatible, and robust. Their transparency facilitates the study of any cellular response such as calcium oscillation and the fluorescent observation of an immunostained cytoskeleton, as exploited in the Si/SiO_2_ micro-rolls used for studying yeast cell behaviour^53^. The biocompatible micro-rolls in this study neither require cytotoxic etchant nor suffer from the stochastic encapsulation of cells that voluntarily migrate in, unlike conventional self-folded, inorganic cylinders^2,3,62^. This is attributable to the non-cytotoxic removal of the sacrificial hydrogel layer (Ca-alginate), which enables the batch encapsulation of multiple cells. The combination of more than two types of transparent polymers in the bilayer should be both compatible with the underlying Ca-alginate, modifiable with an appropriately coated appropriate ECM, and resistant to chemical etching in the fabrication process. As these materials, we specifically selected silk fibroin and parylene-C as the transparent polymer with heterogeneous high elastic moduli, approximately 3 GPa and 80 MPa, respectively (Supplementary Information 2–3). Weak adhesion in layered hydrogel film also makes it difficult to scale down to the nano/micro level and limits the generation of stiffness gradients, which compelled us to induce a stress gradient in homogeneous films. The micro-rolls proposed in this study were improved by coating the film with parylene using CVD, which resulted in the fabrication of single-cell-sized 3D structures with highly precise geometry in the same manner as bimetal films. If the micro-rolls meet the above requirements, they will not be limited as regards type of polymeric material, for instance, SU-8^16^, poly(*N*-isopropylacrylamide)^13,14^, polycaprolactone^10^, or poly(ethylene glycol)^26^ could be used. Furthermore, our self-folded cylindrical 3D structures contained few wrinkles or deflections, and we were able to produce a fibre-shaped cellular morphology and promote the correct cellular functions.

The biocompatibility of silk fibroin and parylene also ensured the long-term encapsulation, culture (>1 month), and manipulation of encapsulated adherent cells without cytotoxicity, in a similar manner to conventional self-folded 3D hollow structures made of metals for culturing adherent cells. Importantly, the adherent cells inside the micro-rolls can be stably encapsulated and manipulated while retaining their adhesive properties. Although the manipulation of cell-laden micro-rolls demonstrated here was entirely manually operated, our platform could be incorporated with an encapsulated magnet-responsive layer^44,63^ or a microfluidic system^64^ in order to achieve fast, reliable, and automated manipulation^65,66^. The distance-mediated interconnection of elongated neurites between more than two neuron-laden micro-rolls is worth of a detailed investigation involving optimising their sizes, location, and patterns from the perspective of building synthetic neural circuits and tissue engineering^54–56^. By harnessing this high handleability, the assembly and reconstruction of highly oriented primary neural cells could be achieved. In future, this method could be expanded to reconstruct tissues with various shapes *in vitro* that are suitable for exploring single-cell assays, tissue engineering, and implantable *ex vivo* tissue grafts.

## Methods

The methods are described in full in Supplementary Information.

### Material preparation

We used silk fibroin hydrogel, parylene, and Ca-alginate as biocompatible materials for micro-roll fabrication. Dichloro-di(*p*-xylylene) (parylene, DPX-C) powder was purchased from Speedline Technology, USA. Aqueous silk fibroin solution was produced from the cocoons of *Bombyx mori* (*B*. *mori*) silkworms. Briefly, the silk cocoons (Satoyama Craft, Japan) were degummed for 30 min in a boiling 20 mM solution of sodium carbonate to remove silk sericin protein and then rinsed with distilled water. The extracted silk fibroin fibres were dried overnight and then dissolved in a 9.3 M lithium bromide solution (Tokyo Chemical Industry Co., Ltd. Japan) for 4 h. This was followed by a dialysis step with Spectra/Por^®^ Dialysis Tubing (MWCO 3500 K, Spectrum, Inc., USA) to convert the solvent to distilled water, yielding a 40 mg/mL solution of silk fibroin. Alginate sodium salt (A2033, Sigma, USA) was dissolved in distilled water to form an aqueous solution of sodium alginate. A positive photoresist (S1813G) and MICROPOSIT 351 developer were purchased from Rohm and Haas (USA).

### Cell preparation and suspension

Chinese hamster ovary (CHO), human embryonic kidney (HEK), human foreskin fibroblast (HFF), and human hepato-cellular carcinoma (Huh-7) cell lines were purchased from DS Pharma Biomedical, Japan. The cells were cultured in Dulbecco’s modified Eagle’s medium (DMEM, ThermoFisher, USA) containing 10% foetal bovine serum (FBS) supplemented with 1% L-glutamine and 10 μg/ml gentamicin (Invitrogen, USA). All the cells were maintained at 37 °C in a humidified incubator containing 5% CO_2_.

Primary neuronal cells and cardiomyocytes were prepared from the hippocampi and hearts of 18th day embryo (E18) Wistar rats. The hippocampi were trypsinized, dissociated with 0.5 mg/mL trypsin (Gibco, Thermo Fisher Scientific Inc., USA) for 10 min, and mechanically triturated. The cell suspension was washed in centrifuges and filtrated, and then the cells were plated onto micro-rolls at the indicated initial densities. The hippocampal cell cultures were maintained in supplemented Neurobasal medium (Gibco) with the addition of 0.5 mM glutamine (Sigma-Aldrich, USA), 25 μM glutamate (Sigma-Aldrich), 50 μg/mL gentamicin (Gibco), and 2% B-27 supplement (Gibco). The cardiac tissues were trypsinised, dissociated by 0.05% trypsin-EDTA (Gibco) for 30 min, and inhibited with 0.05% trypsin inhibitor (Gibco). The cardiomyocyte cultures were maintained in DMEM supplemented with the addition of 5% horse serum (Gibco), 5% foetal bovine serum (Gibco), 2.5 μg/mL insulin (Sigma-Aldrich), 100 U/mL penicillin, and 100 μg/mL streptomycin (Sigma-Aldrich). The cells were maintained in a 5% CO_2_ incubator at 37 °C with saturated humidity. All experimental procedures involving animals were approved by and conducted in accordance with the Institutional Animal Care and Ethics Committees of Nippon Telegraph and Telephone Corporation (NTT) and NTT Science and Core Technology Laboratory Group (experimental protocol no. RIN2014-04), which are in compliance with the Guidelines for the Proper Conduct of Animal Experiments of the Science Council of Japan (kohyo-20-k16-2, 2006).

### Micro-roll fabrication

Supplementary Fig. S1 shows the process we used to fabricate micro-sized micro-rolls. A 2 wt% solution of sodium alginate was spin-coated on either silicon wafers or SiO_2_ substrates at a maximum speed of 3,000 r.p.m. for 30 s. The substrates were then immersed in 100 mM calcium chloride (Wako Pure Chemical, Japan) to form an alginate hydrogel layer with Ca^2+^ ions. The silk fibroin solution was drawn up by a syringe with an 18-G needle and spin-coated on the surface of an alginate hydrogel layer at various speeds for 30 s. The result was immersed in methanol (Kanto Chemical, Japan) for 2 days to produce silk fibroin hydrogel layers that were 50–400 nm thick. When the solution was not drawn through a syringe, the micro-rolls began to refold in the opposite direction and became flat. Then, a layer of parylene 50–500 nm thick was deposited using a chemical deposition system (LABCOATER PDS2010, USA). Inside the deposition system, parylene was first vaporized at 150 °C, and then pyrolysed at 690 °C to generate chloro-*p*-xylylene monomer. A reduction in the chamber temperature caused chloro-*p*-xylylene to condense onto the silk fibroin layer to form parylene membranes. The parylene-C thickness was controlled by controlling the weight of the initially loaded dichloro-di(*p*-xylylene) at a rate of approximately 0.625 μm/g. After the parylene deposition, photoresist was spin-coated, exposed to ultraviolet light through chromium photo-masks, and developed in MICROPOSIT 351. Then, the triple-layered film composed of alginate hydrogel, silk fibroin, and parylene was etched with O_2_ plasma (CV-e300, Mory Engineering, Japan) to fabricate a micro-patterned film array. Finally, the residual photoresist was removed with acetone (Kanto Chemical, Japan). The array was exposed to UV light for 5 min for sterilization and stored in a vacuum desiccator prior to use.

## Electronic supplementary material

Supplementary Information

Supplementary Movie SM1

Supplementary Movie SM2

Supplementary Movie SM3

Supplementary Movie SM4

Supplementary Movie SM5

Supplementary Movie SM6

Supplementary Movie SM7
