# Supplementary material for: Cell Assembly in Self-foldable Multi-layered Soft Micro-rolls
Source: Sci Rep. 2017 Dec 22;7:17376. doi: 10.1038/s41598-017-17403-0 (PMC5741765; doi:10.1038/s41598-017-17403-0)
Supplement: Supplementary file 1 — Supplementary Information [file 41598_2017_17403_MOESM1_ESM.pdf]

Supporting Information

Cell Assembly in Self-foldable Multi-layered Soft Micro-rolls

Authors:

Tetsuhiko F. Teshima, Hiroshi Nakashima, Yuko Ueno, Satoshi Sasaki, Calum S. Henderson†, Shingo Tsukada

Affiliations:

NTT Basic Research Laboratories, NTT Corporation, Japan

† Present address: School of Chemistry, The University of Edinburgh, Scotland

David Brewster Road, Edinburgh EH9 3FJ, United Kingdom

Table of Contents:

1. Materials and Methods

(**Supplementary Figure S1**)

2. Supplementary experimental results and figures

2-1. FIB-sectioned SEM cross-sectional image of multi-layered films
(**Supplementary Figure S2**)

2-2. Fluorescent observation of Qdot-particle-encapsulating films
(**Supplementary Figure S3**, **Supplementary Movie SM1**)

2-3. Theoretical calculation of curvature radius of micro-rolls
(**Supplementary Figure S4**)

2-4. Various 3D geometries of self-folded micro-rolls
(**Supplementary Figure S5**, **Supplementary Movie SM2**)

2-5. Cell encapsulation and culture inside micro-rolls
(**Supplementary Figure S6**, **Supplementary Movie SM3 and SM4**)

2-6. Manipulation of cell-laden micro-rolls
(**Supplementary Figure S7**, **Supplementary Movie SM5**)

2-7. Optical images and time-dependent displacement of encapsulated beating cardiomyocytes
(**Supplementary Figure S8**, **Supplementary Figure S9,**
Supplementary Movie SM6, **Supplementary Movie SM7**)

3. Supplementary references

1. Materials and Methods

Fabrication of micro-rolls. Supplementary Fig. S1 shows the four-step self-foldable micro-roll fabrication process in detail. The first step is to form a triple-layered polymeric film. A 2 wt% solution of sodium alginate was spin-coated either on silicon wafers with a thermally oxidised film surface (Canosis Co., Ltd., Japan) or on a SiO_2 substrate (NEO cover glass, 0.12 to 0.17 mm thick, Matsunami Glass IND., Japan) at a maximum speed of 3,000 r.p.m. for 30 s, and immersed in 100 mM calcium chloride (Wako Pure Chemical, Japan) to form an alginate hydrogel layer with Ca^{2+} ions (Ca-alginate) (S1a). The silk fibroin solution was subsequently drawn up by a syringe with an 18-G needle and spin-coated on the surface of the alginate hydrogel layer at various speeds for 30 s, and then immersed in methanol (Kanto Chemical, Japan) for 2 days to produce silk fibroin hydrogel layers 50 – 400 nm thick (S1b). In the absence of drawing through a syringe, the micro-rolls began to refold in the opposite direction, and they became flat because of the insufficient gelation of the silk fibroin. Then, a layer of parylene 50 – 500 nm thick was deposited using a chemical deposition system (LABCOTER PDS2010). Inside the deposition system, parylene was first vaporised at 150°C, and then pyrolysed at 690°C to generate chloro-*p*-xylylene monomer. A reduction in the chamber temperature caused chloro-*p*-xylylene to condense onto the silk fibroin layer to form parylene membranes (S1c). The parylene-C thickness was controlled by controlling the weight of the initially loaded dichloro-di(*p*-xylylene) at a rate of approximately 6.25×10^{-1} μm/g. To visualize each layer of the trilaminar film, Qdots with red and green fluorescence were embedded in the Ca-alginate and the silk fibroin layer, respectively. In the second step, the negative photoresist S1813G was spin-coated on the surface of the deposited parylene-C, exposed to ultraviolet light through chromium photo-masks, and developed in tetramethylammonium hydroxide

(TMAH, MICROPOSIT 351 developer) (S1d). In the third step, the triple-layered film composed of alginate hydrogel, silk fibroin, and parylene was etched with O_2 plasma (CV-e300, Mory Engineering, Japan) to fabricate an array of micropatterned films (S1e, f).[1-3] Finally, the residual photoresist was removed with acetone (Kanto Chemical, Japan) (S1g). The array was exposed to UV light for 5 min for sterilization and stored in a vacuum desiccator prior to use. The cell encapsulation process shown in Supplementary Fig. S1(h-k) is described in detail in Supplementary Information 2-5.

Cell preparation and suspension. Chinese hamster ovary (CHO), human embryonic kidney (HEK), human foreskin fibroblast (HFF), and human hepato-cellular carcinoma (Huh-7) cell lines were purchased from DS Pharma Biomedical, Japan. The cells were cultured in Dulbecco's modified Eagle's medium (DMEM, ThermoFisher, USA) containing 10% foetal bovine serum (FBS) supplemented with 1% *L*-glutamine and 10 μg/ml gentamicin (Invitrogen, USA). All the cells were maintained at 37°C in a humidified incubator containing 5% CO_2. Once the cells were confluent, they were trypsinised with 0.025% trypsin in 0.01% ethylene-diaminetetraacetic acid (EDTA, Sigma-Aldrich, USA) solution and passaged at a subculture ratio of 1:5. The harvested adherent cell lines were released from the culture dishes by adding trypsin; they were then loaded into a microtube and centrifuged. The collected cells were re-suspended by gently pipetting them into a Petri dish and then allowing them to settle and grow in an incubator.

Primary neuronal cells and cardiomyocytes were prepared from the hippocampi and hearts of 18th day embryo (E18) Wistar rats. The hippocampi were trypsinised, dissociated with 0.5 mg/mL trypsin (Gibco, Thermo Fisher Scientific Inc., USA) for 10 min, and mechanically triturated. The cell suspension was washed in a

centrifuge and filtrated, and then the cells were plated onto micro-rolls at the indicated initial densities. The hippocampal cell cultures were maintained in supplemented Neurobasal medium (Gibco, Thermo Fisher Scientific Inc., USA) with the addition of 0.5 mM glutamine (Sigma-Aldrich, USA), 25 μM glutamate (Sigma-Aldrich, USA), 50 μg/mL gentamicin (Gibco, Thermo Fisher Scientific Inc., USA), and 2% B-27 supplement (Gibco, Thermo Fisher Scientific Inc., USA). The cardiac tissues were trypsinised, dissociated by 0.05% trypsin-EDTA (Gibco, Thermo Fisher Scientific Inc., USA) for 30 min, and inhibited with 0.05% trypsin inhibitor (Gibco, Thermo Fisher Scientific Inc., USA). The cardiomyocyte cultures were maintained in DMEM (Gibco, Thermo Fisher Scientific Inc., USA) supplemented with the addition of 5% horse serum (Gibco, Thermo Fisher Scientific Inc., USA), 5% foetal bovine serum (Gibco), 2.5 μg/mL insulin (Sigma-Aldrich, USA), 100 U/mL penicillin, and 100 μg/mL streptomycin (Sigma-Aldrich, USA). The cells were maintained in a 5% CO_2 incubator at 37ºC with saturated humidity.

Characterization of micro-rolls. The film thickness was measured using a surface profiler (Alpha Step IQ, KLA-Tencor, USA) with a resolution of 0.1 nm. The thickness of each layer was controllable by controlling the concentration, viscosity, and spin-coating speed. The film was spectroscopically characterized before or after self-folding by using an FTIR spectrometer (VERTEX 80, Bruker Corp., USA) and a Raman spectrometer (inVia Qontor, Renishaw, UK).

Microscopic imaging of cell-laden micro-rolls. To assess cell viability, living and non-living cells were dyed with calcein-AM solution (Invitrogen, USA) and ethidium homodimer-1 (EthD-1, Molecular Probes, USA) following the manufacturer's protocol

for live/dead assay. For immunostaining, the hippocampal cells were grown for 1 week, fixed with 4 wt% paraformaldehyde (P6148, Sigma Aldrich, USA) for 15 min, permeabilised with 0.5% Triton X-100 (T9284, Sigma Aldrich, USA) for 3 min, and incubated in 1% bovine serum albumin (BSA, Sigma Aldrich, USA) overnight at 4°C. Immunostaining was carried out as follows: actin fibres were probed with Alexa Fluor 594-conjugated phalloidin (A12381, Invitrogen, USA); somatic, nuclear, dendritic, spine, and axonal proteins distributed across the pan-neuronal cytoarchitecture were visualized with Alexa Fluor 488-conjugated Pan Neuronal Marker monoclonal antibody (MAB2300X, Merck Millipore, USA).

Bright-field, phase contrast, and fluorescence images were obtained using 10×, 20×, and 40× objectives, an inverted optical microscope with phase contrast (ECLIPSE TE2000, Nikon, Japan; BZ-X700, Keyence, Japan), a charge-coupled device camera (DP30, DP73, Olympus, Japan) and imaging software (MetaMorph, Molecular Devices, USA; cellSens Dimension, Olympus, Japan). Cross-sectional *z*-stack images of the cells inside the micro-rolls were recorded using an inverted confocal laser-scanning microscope (LSM 510, Carl Zeiss, Germany) and the installed imaging software (ZEN2009). Time-lapse images of the cell behaviour inside the micro-rolls were captured using a folded optical structured microscope system (BZ-X700, Keyence, Japan) with a stage top incubator (INU-KIW, TOKAI HIT, Japan).

Fast Fourier transform (FFT) analyses were conducted on selected areas of the fluorescence images of immunostained cytoskeletons within and outside the micro-rolls. The images were converted to greyscale pixels (1024 ×1024, 256 × 256 pixels), and transformed with an ImageJ plug-in Hanning window and FFT (provided by National Institutes of Health (NIH)). The degree of stained cells and their cytoskeleton reflects on the distribution in a pattern. A graphical depiction of the FFT frequency

distribution was transferred in order to place a circular projection on the FFT output image and conduct a radial summation of the pixel intensities for one degree increment from 0 to 180 with the Image-J plug-in Oval Profile. Supplementary Fig. 9d and 9e were obtained by summing the pixel intensities encountered along the specific radius in the FFT output image. To quantify the alignments of cytoskeletons and neurites, the texture direction index was calculated from the amplitude of the summed radial intensities along a line from the centre to the edge of the FFT image.

SEM observation. A substrate with cell-laden micro-rolls was gently rinsed with PBS and then fixed with 2 wt% glutaraldehyde (Wako, Japan) for 30 min. The samples were then dehydrated with a series of ethanol/water mixtures of increasing ethanol density (30 - 99%). After immersing the samples in *tert*-butyl alcohol (Wako, Japan), they were lyophilised using a freeze drier (FS-2030, EYELA, Japan). The fixed cells were then sputter coated with gold (Ion Sputter E-1030; Hitachi, Japan) and observed with an FIB-SEM (Auriga 60 Cross Beam Workstation; Carl Zeiss, Germany).

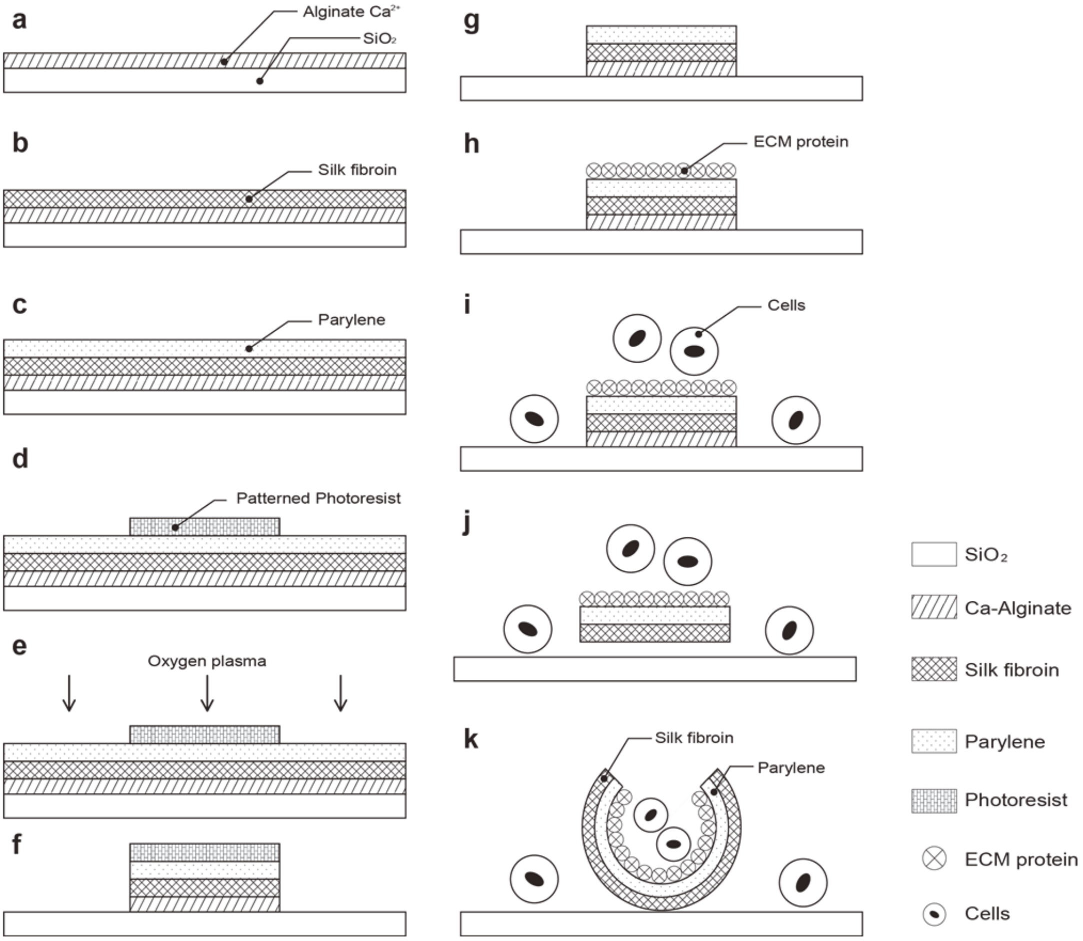

Supplementary Figure S1. **Process for fabricating self-folded micro-rolls for encapsulating and culturing cells.** **a**, Sodium alginate was spin-coated and gelated to Ca-alginate on a SiO_2 substrate. **b**, Silk fibroin solution was spin-coated and gelated on the Ca-alginate film. **c**, Parylene-C was deposited on the silk fibroin layer. **d**, The photoresist was spin-coated and micropatterned on the surface of parylene-C. **e-g**, The triple-layered film was etched with O_2 plasma through a micropatterned photoresist mask. **h**, The parylene surface was modified with ECM proteins. **i**, The cells were suspended on the parylene layer. **j**, The Ca-alginate layer was removed by adding EDTA. Then, the films autonomously self-folded into cylindrical shapes. **k**, After the sacrificial layer had been released in a non-cytotoxic process, the self-folded cylindrical structures encapsulated the cells.

2. Supplementary experimental results and figures

2-1. FIB-sectioned SEM cross-sectional image of multi-layered film

To investigate the atomic composition of the film and micro-rolls using energy dispersive X-ray (EDX) analysis, we performed focused ion beam (FIB) cross-sectioning. The cross-section was polished and milled with a Dual-beam (Auriga 60 CrossBeam, Carl Zeiss, Germany) equipped with a Nanometre Pattern Generation System (JC Nabity Lithography Systems, USA). Before the FIB sectioning, the samples were coated with sputtered Au. The films were milled with a 4 nA beam current at an ion voltage of 30 kV, and to a depth of 3 μm. The transversal cross-sectional image shows five laminated layers composed of solid triple-layered polymeric films and a Si substrate with oxidised SiO_2 film (Supplementary Fig. S2a,b). There were no gaps or bubbles between the layers. The granular particles on the parylene and SiO_2 surface were Au atoms sputtered to avoid any increase in the electron charge. We also performed FIB cross-sectioning of self-folded micro-rolls after the folding process using an ion voltage of 30 kV and a current of 1 nA (Supplementary Fig. S2c). The SEM cross-section image in Supplementary Fig. S2d shows that two layers composed of parylene and silk fibroin were curved in conjunction without any bilayer separation after the folding process. We also found that the outer surface of the silk fibroin had fewer wrinkles and less deformation than that caused by folding the thin film.

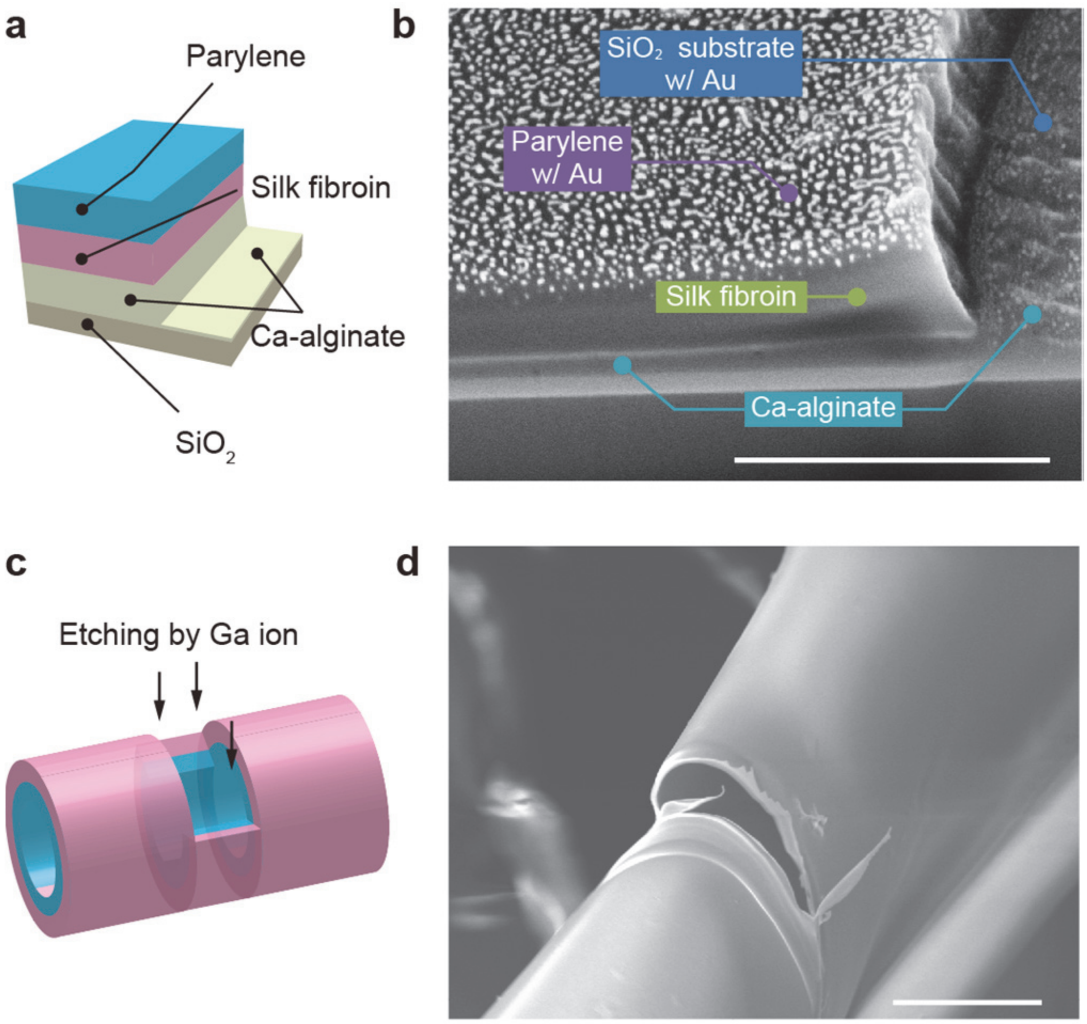

Supplementary Figure S2. **FIB-sectioned triple-layered film on substrate.** **a**, **b**, Schematic and SEM image depicting FIB-sectioned micropatterned triple-layered film composed of alginate hydrogel, silk fibroin, and parylene on a SiO_2/Si substrate (tilt 45°). **c**, **d**, Schematic and SEM image of partly FIB-milled micro-rolls (tilt 45°). Scale bars: (**b**) 1 μm, (**d**) 10 μm.

2-2. Fluorescent observation of Qdot-particle-encapsulating films

To visualize each layer inside the micro-rolls, we embedded red and green fluorescent Qdot nano-particles (Qdot 525/655 ITK Carboxyl Quantum Dots, Gibco, Thermo Fisher Scientific Inc., USA) in Ca-alginate and silk fibroin. Parylene-C

exhibits weak auto-fluorescence when excited with ultraviolet light.[4,5] The fluorescence images in Supplementary Fig. S3a show that micro-patterned square films were formed by laminating parylene (blue), silk fibroin (green), and Ca-alginate (red) once the photolithographic process has been completed. O_2 plasma etching completely removed every part of these three layers that was not covered with a photoresist mask. After adding the EDTA, the fluorescent images indicated the deformation of each layer. The red fluorescence images show the interference fringes that emerged from the film edge, causing deflections and wrinkles with the Ca-alginate film (Supplementary Fig. S3b). Once the fringes had disappeared, the fluorescence intensity of the green fluorescent Qdot and the autofluorescence of parylene (blue) increased, which indicates the simultaneous deformation of the stably adhesive bilayer (Supplementary Fig. S3c). In contrast, the average fluorescence intensity of the Ca-alginate (red) decreased as the folding steps proceeded. Although the dissolution speed depended on the ambient concentration of the EDTA, the Ca-alginate layer was also not totally dissolved, and some of it remained on the surface of the SiO_2 and silk fibroin layers at any EDTA concentration, partly due to the non-specific adsorption of the nano-particles. This is because SiO_2 surfaces immersed in water have a negative surface charge density,[6] before and after the folding process.

The self-folding yield decreased with a longer swelling time after the micro-rolls were immersed in DMEM solution (Supplementary Fig. 1e). More than 20 min after immersion in DMEM, the dissolution of the alginate hydrogel layer reduced the self-folding success rate to less than 5%. The slow speed of dissolution of Ca-alginate resulted in film transformation failure, and caused the film to remain flat, partly because the self-folding process was driven by the swelling of the silk fibroin. The low self-folding yield caused wrinkles or creases to appear on the film surface.

This is partly because the film being restrained at the two surfaces did not result in internal stress in the thickness direction due to the longer immersion. Since the driving force behind self-folding is the change in the volume of the swelling silk fibroin, the fact that the internal stress is unchanged leads to the released film remaining flat. Therefore, it is necessary to release the films within 10 min of placing them in wet conditions.

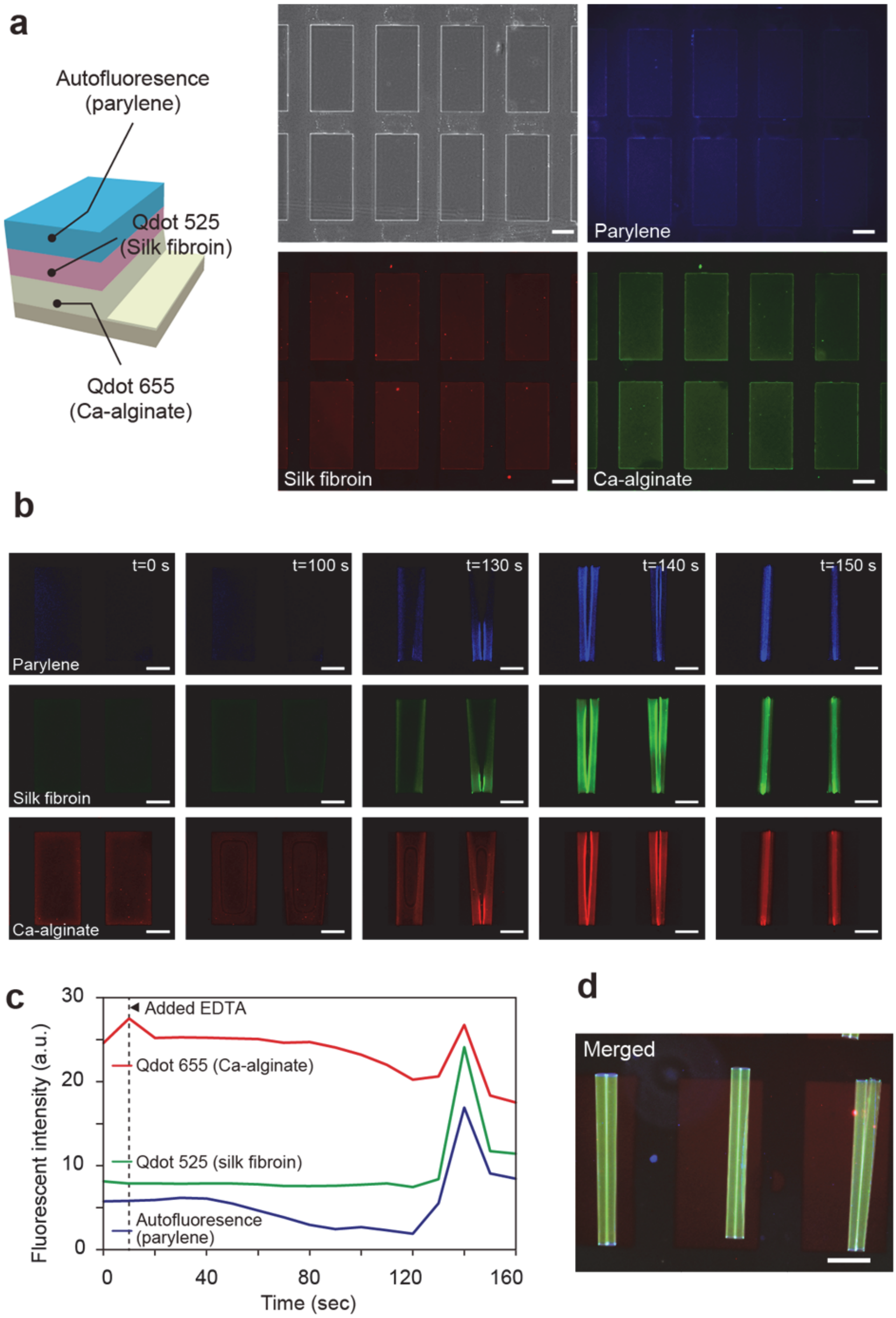

Supplementary Figure S3. **Visualization of each layer in film and micro-rolls. a**, An explanatory illustration (left), phase-contrast, and fluorescence images (right) of multiple layers visualised by using embedded fluorescent nano-particles. Parylene: (autofluorescent blue), Ca-alginate: red (Qdot 655), silk fibroin: green (Qdot 525). **b**, Time-lapsed fluorescence images showing sequential self-folded micro-rolls. Removal of the sacrificial Ca-alginate layer triggered the spontaneous self-folding of

micro-rolls within 150 sec. **c**, Time-dependent plot of average fluorescence intensity in 200×400 μm^2 film area. Ca-alginate: red line, silk fibroin: green line, parylene: blue line. **d**, A merged fluorescence image of micro-rolls. The scale bars are 100 μm.

Supplementary Movie SM1. Time-lapse phase-contrast and fluorescent observation of self-folding process. Parylene: (autofluorescent blue), Ca-alginate: red (Qdot 655), silk fibroin: green (Qdot 525). The movies are played at 2× normal speed.

2-3. Theoretical calculation of curvature radius of micro-rolls

Various microstructures can be produced by modifying the 2D geometrical design of micro-patterned film. Here, a quantitative prediction of the effects of the 2D micropatterns on the final 3D geometry of the micro-rolls after self-folding is essential in terms of creating the predictive structure–function relationships for bending bilayer composites of silk fibroin and parylene. The films are autonomously self-folded into cylindrical shapes based on the different strain gradients within the nano-thick films. In the absence of external forces, the forces exerted on the cross-section of the film are in equilibrium. We measured the curvature radii of micro-rolls with a closed cross-sectional circle loop by halving the distance between two edges in images obtained by upright microscopy (VHX-5000, KEYENCE, Japan). In contrast, we estimated the curvature radius (ρ) of micro-rolls with an open cross-sectional arc loop using the Newton-Raphson method. We measured the arc length (L_a) and chord length (L_c) from images of thin films obtained before and after folding, and calculated the central angle (θ) and curvature radius (ρ) of the micro-rolls by employing $L_a=\rho\cdot\theta$ and $L_a=2\rho\cdot\theta\cdot\sin(\theta/2)$. The parylene thickness was measured by using parylene films

deposited on the SiO_2 surface and profiling the gap surface of the films with a step profiler (Alpha Step IQ). The swelling ratio of silk fibroin was measured by immersing 3.3 mg and 0.33 mg of gelated silk fibroin with embedded red fluorescent Qdot nano-particles for change of weight and volume, respectively (Qdot 655). The changes of weight and volume of silk fibroin hydrogel immersed in distilled water were measured with the electronic balance and fluorescence microscope, respectively. Supplementary Fig. Sb shows the time-dependent change in weight and volume of swollen silk fibroin pallets. While the silk fibroin hydrogel absorbed water until its weight becomes more than twice, the saturated volume change of hydrogel was estimated to be less than approximately 15% in equilibrium.

Spin-coating at 1000 and 3000 rpm formed silk fibroin films with thicknesses of approximately 210 nm and 100 nm, respectively. Parylene film with a thickness (t) of 0.97 μm and width (w) of 20 mm, and silk fibroin film with a thickness (t) of 25.2 μm and width (w) of 4.5 mm were used for mechanical testing, respectively. They were fabricated on a silicone rubber substrate, peeled from it, and cut into dog-bone shapes that were 50 mm long. The dog-bone shaped films were then glued to two paired rigid clamps with inter-marked line distance of 10 mm (L_0). The force (F) – elongation (l) curves with toughness modulus and elongation at a break were obtained from tensile stress versus engineering strain curves by using a table-top tension/compression testing device (STB1225S, A&D Company, Japan) equipped with 50 N load cells and analysis software (Supplementary Fig. S4c). Supplementary Fig. S4d and S4e show the force–elongation curves of parylene and swollen silk fibroin hydrogel, respectively. Then, the elastic modulus (E) was calculated from the linear area in the stress–strain curves as follows:

$$E = \frac{\sigma}{\varepsilon}$$

where σ and ε is the stress and strain of the elongated films, respectively. The expansion of the parylene layer in the bilayer was negligible, when it was soaked in water. Then, we set the following parameters: E_p=3450 MPa, E_s=80 MPa, and ε_p=0.4.[7-9] Because the protein network obeys the law of rubber elasticity, we assumed ε_s to be a Poisson ratio of 0.5.[10-12] The corresponding values of ρ and t_p, and the theoretical curve are plotted in Fig. 2d, 2e, and Supplementary Fig. S4f-g, respectively.

We investigated the influence of the film geometry on the final curvature (ρ) of self-folded micro-rolls after swelling in water with the help of the bimetallic theory (equation 1).[13] Supplementary Fig. S4f shows the plots of the average curvature radius of free-floating self-folded 200×400 μm^2 films with silk fibroin layer thicknesses t_s=210 and 100 nm versus various parylene layer thicknesses (t_p). Compared with the films with t_s=210 nm, that with t_s=100 nm had a larger curvature radius because the volume change of the silk fibroin layer decreased. Thus, the curvature radius ρ was found to be strongly affected by the thicknesses of both parylene and silk fibroin, but the experimental points and Equation (1) agreed less well when the parylene layer was approximately three times thicker than the silk fibroin layer. Supplementary Fig. S4g shows the plots of the average radius of curvature of self-folded films with different longer film lengths (l) and a constant width (w=200 μm) and thickness (t_p=39 nm, t_s=210 nm). This result indicates that ρ is constant at various lengths (l) when w, t_p and t_s are constant. This is partly because the curvature radius of micro-rolls does not depend on l, but only on the cross-sectional geometrical parameters including w, t_p and t_s. This characteristic is helpful for fabricating a scaffold for encapsulated cells with a well-controlled geometry including diameter and tube length as shown in Fig. 3f (400 μm long) and Fig. 4a (20 mm long). Our experimental data follow the trend predicted by Timoshenko's theory (Equation 1) for bimetallic thermostats, if we assume that t_p is

less than 200 nm. However, the elastic modulus of silk fibroin is lower than those of parylene and metals that are used as bimetallic bilayers, resulting in a high tendency for there to be errors in the experimental plots and divergence between the experimental and theoretical radii-based on the metallic bilayers. The ability to predict the final 3D shape of the micro-rolls based on their initial geometrical features makes this a powerful approach for achieving reconstructed cell aggregates with a tailored and programmable shape.

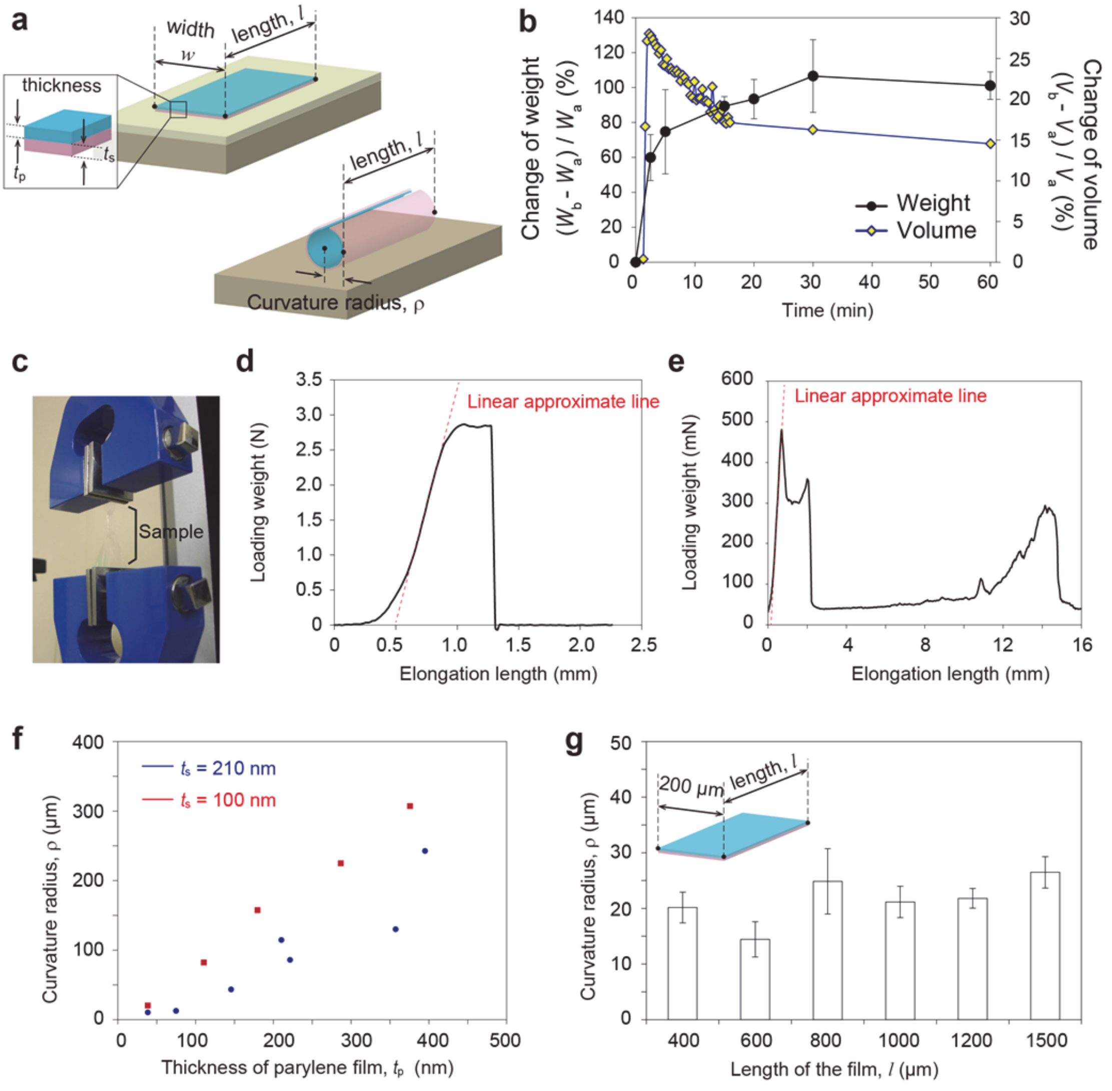

Supplementary Figure S4. **Theoretical calculation of curvature radius of micro-rolls.** **a**, Schematic illustration of length, width, thickness, and diameter of micro-rolls. **b**, Time-dependent swelling kinetics of silk fibroin hydrogel in distilled water at room temperature. W_a and V_a indicate the initial weight and volume before swelling, respectively. W_b and V_b indicate the weight and volume of swollen silk fibroin, respectively. **c**, Photos of parylene film during elongation. **d, e**, Plots of tensile stress versus engineering strain of parylene (d) and swollen silk fibroin (e) measured with a table-top tension/compression testing device. **f**, Plot of average curvature radius of free-floating, self-folded 200×400 μm^2 films with two silk fibroin layer thicknesses (t_s) versus a parylene layer of varying thickness (t_p). **g**, Plot of average radius of curvature of self-folded films with various longer length films (l) and constant width (w=200 μm). The results are shown as the mean ± s.d. of more than eleven separate micro-rolls.

2-4. Various 3D geometries of self-folded micro-rolls

Various 3D cell-laden microstructures can be produced by modifying the 2D geometrical design of the micro-patterned film. Regardless of the shape of the 2D film, the Ca-alginate dissolved at the edge and then proceeded to the centre, and this regulates the bending direction and the final 3D structure as discussed in Supporting Information 2-2 (Supplementary Movie SM2).[14] The multiple joints bridge the rectangular film while connecting them (Supplementary Fig. S5a). Unlike the multi-branched joints (Fig. 2h), straight joints were flexible and easily bent. This ease of bending led to flexibility in the angles of joints in the floating state. We also found that the 2D curved part was more structurally unstable and tended to be folded into straight tube shapes. Accordingly, the structures of the 2D circle "face" areas first became rectangular, and subsequently triangular.

In addition to the rectangular film, film with an array of holes 3 μm in diameter was folded into a cylindrical shape in the same manner as one without holes

(Supplementary Fig. S5b). The 50×400 μm^2 bars bridging the rectangular film connected the cylindrical shapes in a straight line after folding. As shown in Fig. 2h, the rectangles and circles corresponding to arms, limbs, chests, and faces were self-folded and floated. In contrast, the longer arms of the cross portion retained their shape, consequently making it possible to maintain the 3D doll geometry (Supplementary Fig. S5c). The 2D circular "face" area with "eye" and "mouth" holes was not transformed into rectangular or triangular shapes, but folded in a random direction in a different manner from one without holes. An array of holes with a smaller diameter than cells or their neurites (less than 5 μm) will also improve the permeability of O_2 and nutrients for the long-term culture of encapsulated cells as reported previously.[15,16]

In addition to the cylindrical, flower, cross, and doll shapes shown in Fig. 1d and Fig. 2f-h, various other 3D geometries were able to be self-folded thanks to the 2D micropattern design. For instance, meander-shaped films firstly rolled up a part of the curved edges, and finally formed zigzag-shaped tubes (Supplementary Fig. S5d). A lattice film pattern became entangled and formed the structures for wrapping or holding tiny particles when folded (Supplementary Fig. S5e-1, e-2). The structures changed their shapes to caged cylindrical structures with skeletal frames, depending on the parylene thickness (Supplementary Fig. S5e-3). By combining rectangles with structurally stable cross-shaped hinges, a doll-shaped micropattern could be assembled into a higher-order 3D structure. Hence, by incorporating the branched structure in the pattern interior in a variety of 2D shapes, it is possible to produce a more complex 3D shape in addition to cylindrical tubular shapes.

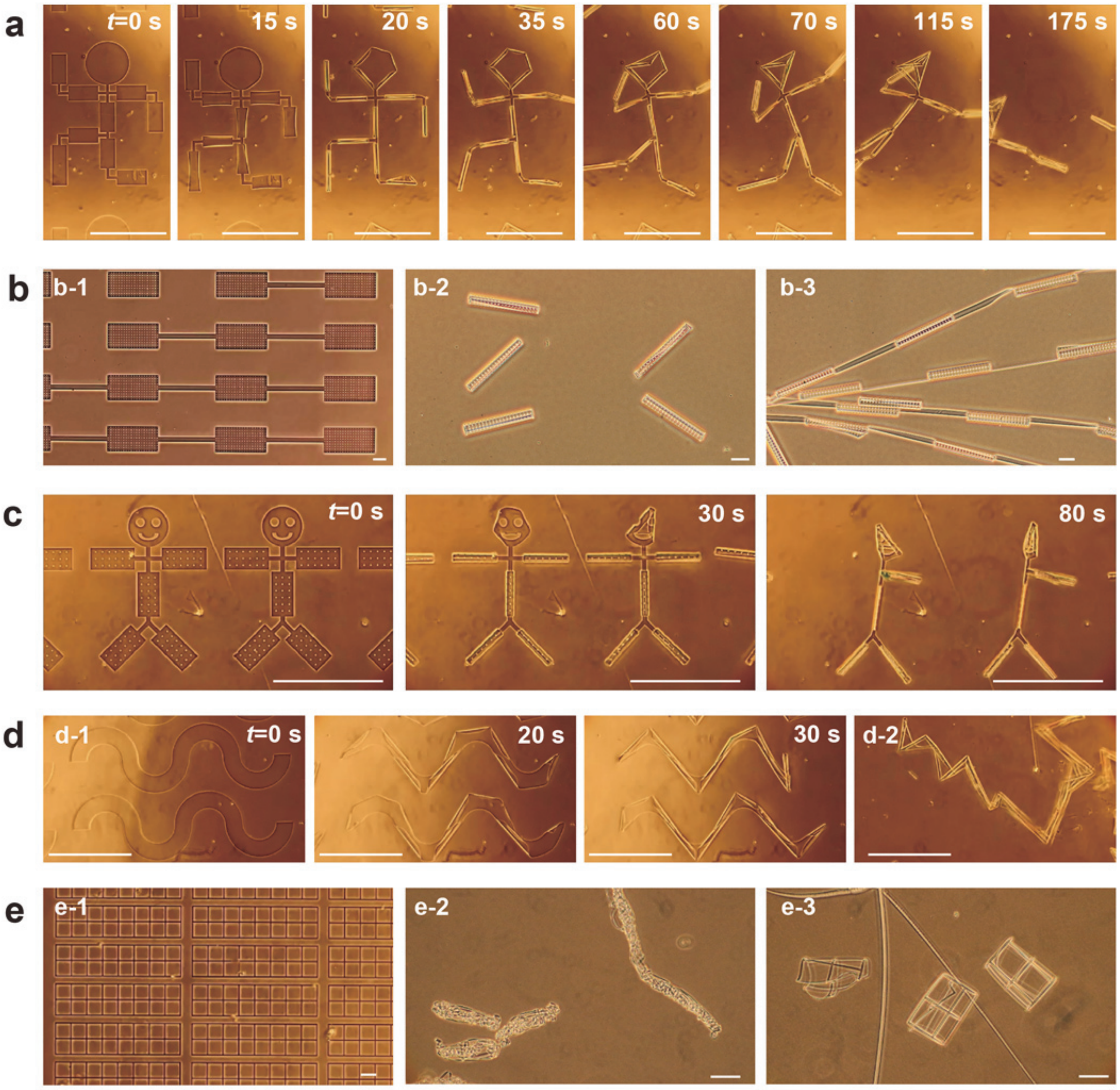

Supplementary Figure S5. **Various 3D geometries of self-folded micro-rolls.** **a**, Time-lapsed sequences of a doll-shaped 3D structure with multiple connecting bars during the folding process. **b**, Microscopic images of a rectangular film with arrayed holes before (b-1) and after (b-2, b-3) folding. They formed tubular structures regardless of the existence of connecting bars. **c**, Time-lapsed sequences of the doll-shaped 3D structure with an array of holes during the folding process. **d**, Series of microscope images showing the self-folding of a meandering 2D micropattern (d-1). A series of wavy shapes were transformed into zig-zag-shaped cylindrical 3D structures. **e**, A latticed pattern of films (e-1) that became entangled (e-2) and skeletal frames of a caged cylindrical structure (e-3), that depended on the parylene thickness. Scale bars: 1 mm in (**a**, **c**, **g**), 100 μm in (**b**, **e**).

Supplementary Movie SM2. Self-folding of doll-shaped 3D structures with multiple connecting bars during the folding process. The movies are played at 4× and 16× normal speed.

2-5. Cell encapsulation and culture inside micro-rolls

As applications of finely manipulable cell-laden micro-rolls, we show the versatility and functionality of cell-laden micro-rolls using various types of cell lines. The cells were encapsulated in the same manner irrespective of cell type. The micro-patterned flat film was immersed in 200 μL of DMEM with 1% fibronectin for 10 min (h). This process modified the parylene surface with ECM protein, thus promoting cellular adhesion. Then, 200 μL of the trypsinised cells was suspended on the film surface at a concentration of 3.0×10^{6} cells/mL (i). The sacrificial Ca-alginate layer coated under the silk fibroin layer was dissolved by adding EDTA to the DMEM solution (j). When 0.5 μL of 0.5 M EDTA solution was added to 200 μL of DMEM (final concentration of EDTA: 1.25 mM), the cells on the film surface were encapsulated inside the micro-rolls within 20 sec and self-folded into a tubular structure (k). Since all the materials constituting the film were transparent, the cells could be observed under both upright and inverted microscopes. Finally, we immediately added 2.5 mL of fresh DMEM for dilution and long-term incubation (final concentration of EDTA: 100 μM). As described in Supporting Information 2-4, the range of 3D geometries was regulated by the design of the 2D micropatterns of the film. Supplementary Fig. S6a and Supplementary Movie SM3 show that these 3D structures, for example doll-shaped patterns encapsulated the CHO cells within their hollow micro-scale cavities. The multi-branched joints bridging the rectangular film were

useful in assembling higher order 3D structures of cell-laden micro-rolls while connecting single micro-rolls.

We investigated the long-term culture of cells inside the micro-rolls by using a cell-adhesive or non-adhesive SiO_2 surface. The culture dishes were treated with poly-*D*-Lysine (PDL) or extracellular matrix (ECM) solution to form a cell-adhesive layer. To culture the cell lines, the dishes were treated with ECM solution, for example laminin and fibronectin. On the other hand, cover slides coated with PDL were used to improve the attachment of neural somas and neurites and cardiomyocytes. Supplementary Fig. S6b shows that the encapsulated CHO cells became attached to the inner surface of the micro-rolls and proliferated along the surface on days 0, 1, and 5. Then, they protruded from both sides of the bottom edge, and migrated to the dish surface coated with ECM. The inner and outer cells migrated independently, and they did not bridge between the bottom substrate and the micro-rolls. Thus, the cell-laden micro-rolls were floating in the cell-culture medium when the outer cells proliferated to the bottom substrate.

We tested the culturing of four types of adherent cell-lines in the micro-rolls with ρ=40 μm: CHO, HFF, HEK, and Huh-7 cells. When incubated for more than 2 days, both CHO and HFF cells became attached and stretched onto the inner surface of the micro-rolls and then proliferated (Supplementary Fig. S6b). Thereafter, they formed hollow tissue-like aggregates. In contrast, both HEK and Huh-7 cells possessed the property of relatively strong cell-to-cell contact. Therefore, they attached to a part of the inner surface, and aggregated. After 1 day of culture, they completely filled the micro-roll cavity. This difference can presumably be attributed to the differences between the cell mobilities of different cell types and affinity to a substrate coated with ECM. Since micro-rolls can be fabricated from 2D

micro-patterns regardless of micro-roll length, we were able to encapsulate and culture cell aggregates with a scale exceeding 1 cm (Supplementary Fig. S6c).

In contrast, glass-bottom culture dishes were treated with 2-methacryroyloxyethylphosphorylcholine (MPC) polymer to form a non-cell-adhesive layer.[1,17] First, an MPC polymer solution was spin-coated on a substrate, and then the substrate was dried in a chamber with an ethanol atmosphere at room temperature for 20 min to form a uniform MPC polymer layer. We baked the coated dishes at 70°C for 4 h to covalently graft the MPC polymer layer to the substrate via a dehydration reaction. Supplementary Fig. S6d shows that the HEK cell-laden micro-rolls were suspended on the surfaces of the MPC-polymer coated dishes. When we cultured cell-laden micro-rolls on an MPC polymer-coated surface, the cells were localized only on the micro-rolls. As they proliferated, the encapsulated cells filled the cavity and protruded from the bottom. The proliferated cells protruding from the micro-rolls exhibited cell-cell adhesion, and remained floating. After 5 days of culture, they migrated onto the outer silk fibroin surface of the micro-rolls, and completely covered it. During cell migration, the micro-rolls were not attached to the MPC polymer-coated dishes, and they were still floating even after 1 week of culture. These results suggest that MPC polymer enabled the surface to repel proteins and prevent cell adhesion within the micro-rolls.

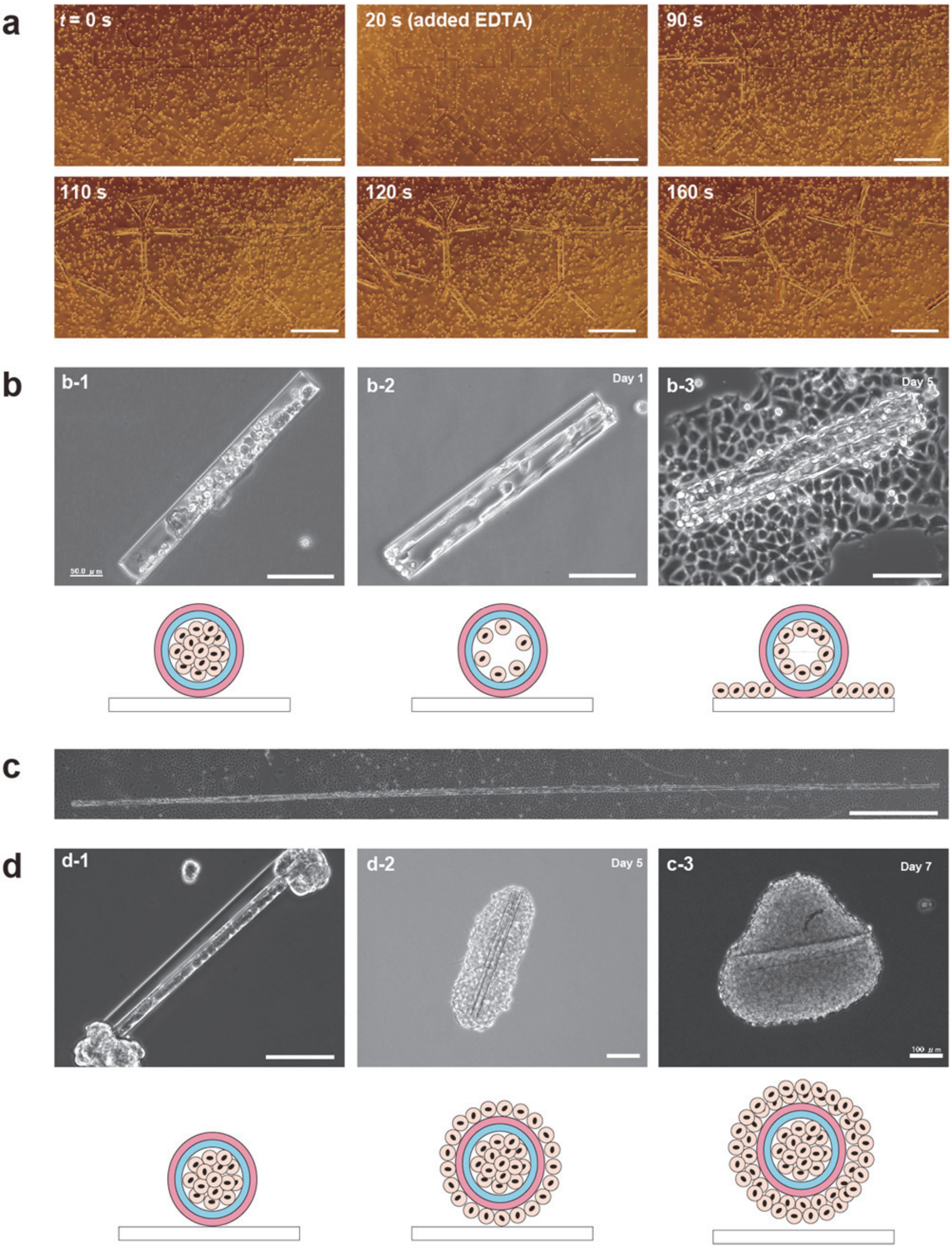

Supplementary Figure S6. Encapsulation and culture of cells within self-folded micro-rolls. **a**, Encapsulation of CHO cells within doll-shaped micro-rolls. **b**, Experimental optical images and schematic images of culture of CHO cells inside micro-rolls on ECM-coated dishes on days 0 (b-1), 1 (b-2), and 5 (b-3). **c**, Centimetre-long micro-rolls including CHO cells. **d**, Experimental optical images and

schematic images of the culture of HEK cells inside the micro-rolls on the MPC polymer-coated dishes on days 3 (c-1), 5 (c-2), and 7 (c-3). Scale bars: (**a**) 500 μm, (**b,d**) 100 μm, (**c**) 100 mm.

Supplementary Movie SM3. Encapsulation of cells within the self-folded micro-rolls. The movies are played at 8× normal speed.

Supplementary Movie SM4. Reconstruction of confocal cross-sectional image of CHO cell-laden micro-rolls. The silk fibroin layer of micro-rolls encapsulating Q-dot 655 nano-sized particles. The cells were stained with calcein-AM for use in live-dead assay. Scale bars are 100 μm.

2-6. Manipulation of cell-laden micro-rolls

The released cell-laden micro-rolls floated in a flow or were able to be manipulated with micro-manipulators, which enabled the collection of specific cells with the desired characteristics and the precise formation of cell-cell contact. The experimental set-up we used to manipulate the released cell-laden micro-rolls while observing them consisted simply of the fabricated device, microscopes with mounted CCD cameras, and a commercially available micromanipulator system (TOPick Pump Single Cell Manipulator, Yodaka Co., Ltd., Japan) (Supplementary Fig. S7a). The straight, *S*-, or *L*-shaped glass capillaries were connected with a picolitre pump, micro-positioners, and a *z*-axis robotic lift actuator (Supplementary Fig. S7b). This manipulation system provided precise control of the location of the glass capillaries, and picolitre amounts of suction and discharge. We acquired images of the manipulated cell-laden micro-rolls with an upright microscope, and displayed them on a monitor

display.

After incubation for more than 1 day, the cell-laden micro-rolls settled at the bottom of the culture dishes without any flow. The introduction of a fluid flow made them float and moved them smoothly. Therefore, micro-rolls with specific cells could be collected, relocated, and rearranged using glass capillaries. Supplementary Movie SM5 and Supplementary Fig. S8c-f show the sequential process for handling cell-laden micro-rolls. This process comprises four procedures: withdrawing the targeted micro-rolls into a glass capillary (inner diameter: 70 μm) (Supplementary Fig. S7c), picking them up from the substrate (Supplementary Fig. S7d), conveying them to different positions, and setting them down in the culture dishes (Supplementary Fig. S7e,f). While the micro-rolls were being conveyed, the withdrawn micro-rolls were located stably inside the glass capillaries. Note that the cells on micro-rolls were manipulated without stripping them from their growth surfaces, which allowed the adhesive state of the manipulated cells to be preserved. During the handling procedure, we did not observe any aggregation of cells in the micro-rolls or in the culture media, thus we achieved the expedient manipulation of single cells. This manipulation system can also be used to transfer cells to different culture dishes, which leads to the formation of co-cultured tube structures composed of different types of cells.

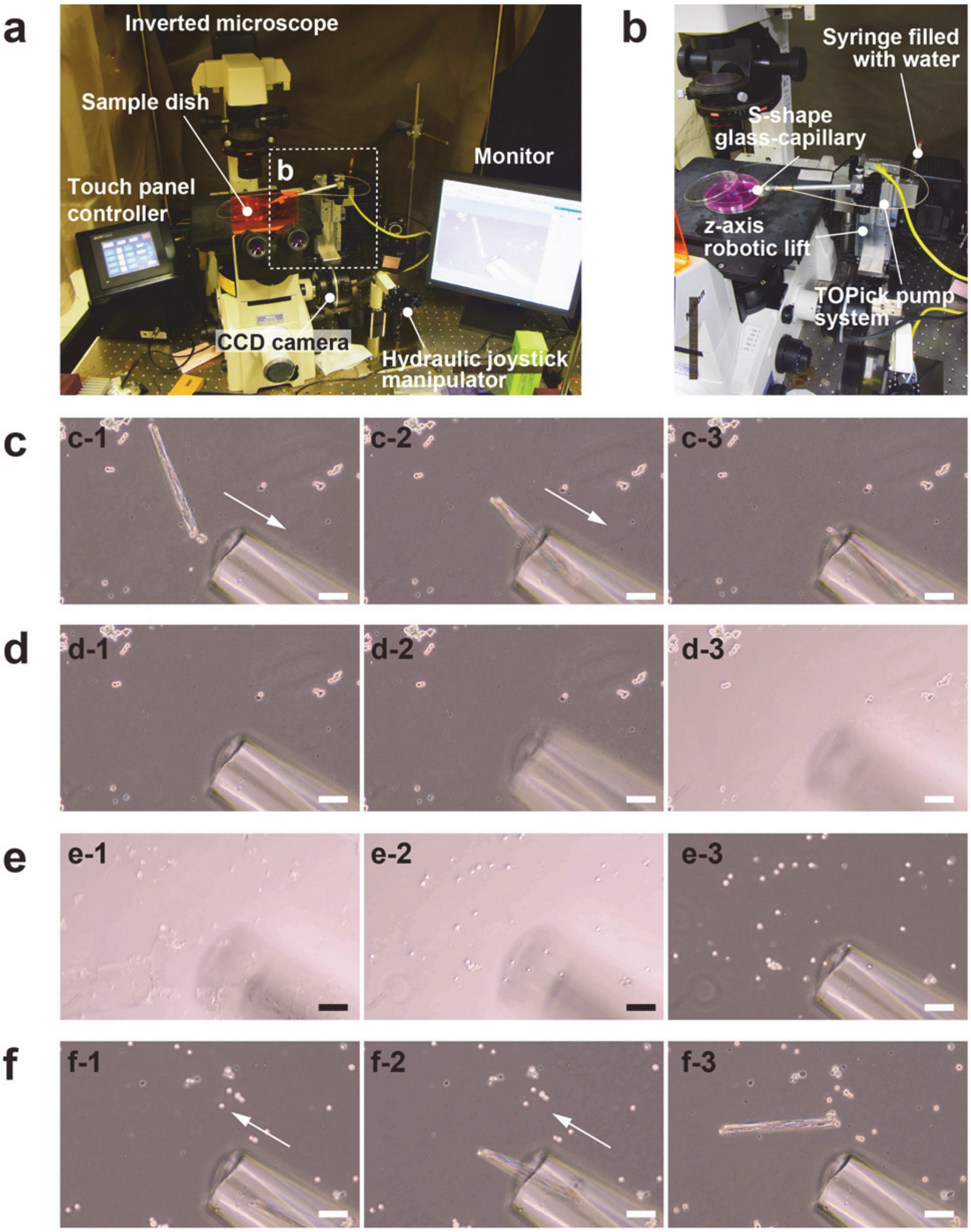

Supplementary Figure S7. Manipulation of cell-laden micro-rolls. a, Experimental set-up of manipulation system installed on microscope with mounted CCD camera. **b**, The glass capillaries were connected to a picolitre pump, micro-positioners, and a *z*-axis robotic lift actuator. **c**, A targeted cell-laden micro-roll was introduced into a glass-capillary (inner diameter: 70 μm) by suction. **d-f**, The micro-rolls were picked up by suction in the atmosphere (d), conveyed to different positions, re-immersed in the medium (e), and they then settled in the culture dishes (f). Scale bars are 100 μm.

Supplementary Movie SM5. Manipulation of cell-laden micro-rolls using glass capillaries. The movies are played at normal or 2× normal speed.

2-7. Optical images and time-dependent displacement of encapsulated beating cardiomyocytes

The beating of cardiomyocytes was traced, and its time-dependent displacement was estimated by using image processing software (ImageJ, NIH, USA) in accordance with a previously reported protocol.[18] The designated area in the captured movie was cropped, and the blurred images caused by vibration were corrected by using the camera shake correction function, "Warp Stabilizer", Adobe Premiere Pro CC (Adobe Systems Incorporated, USA) (Supplementary Fig. S8a). Then, the images were converted to time-lapsed greyscale images. A static background image without beating cells was synthesized by averaging the *z*-projection intensity, and subtracted from a series of images (Supplementary Fig. S8b). After black and white inversion, we designated the intensity threshold and pixel area in order to extract only the motion of the beating cells and remove the motion of objects smaller than cells (Supplementary Fig. S8c). Although the change in the intensity of the white spots does not reflect the displacement distance, its value corresponds to the number of moving cells and the timing of their movements. We estimated the time-dependent displacement by plotting the change in white intensity. Supplementary Movie SM6 shows the movie processed to estimate the displacement of beating cardiomyocytes.

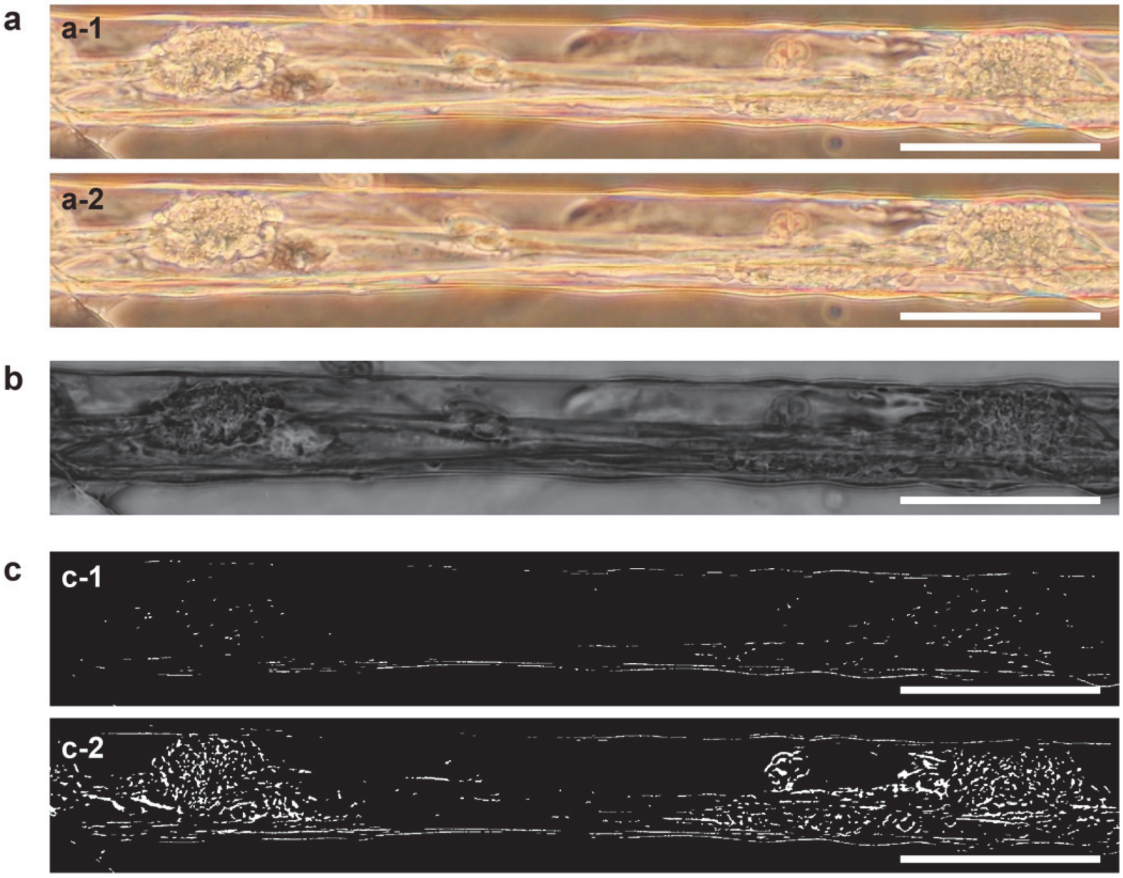

Supplementary Figure S8. **Displacement of beating cardiomyocytes inside micro-rolls.** **a**, Time-lapsed images cropped and corrected with shake correction function. The two images show the cardiomyocytes within the micro-rolls before (a-1) and after beating (a-2). **b**, Static background image synthesized by averaging Z-projection intensity. **c**, Black and white images synthesized by subtracting an averaged background image from the sequential images. The two images shows the cardiomyocytes within the micro-rolls before (c-1) and after beating (c-2). Scale bars are 100 μm.

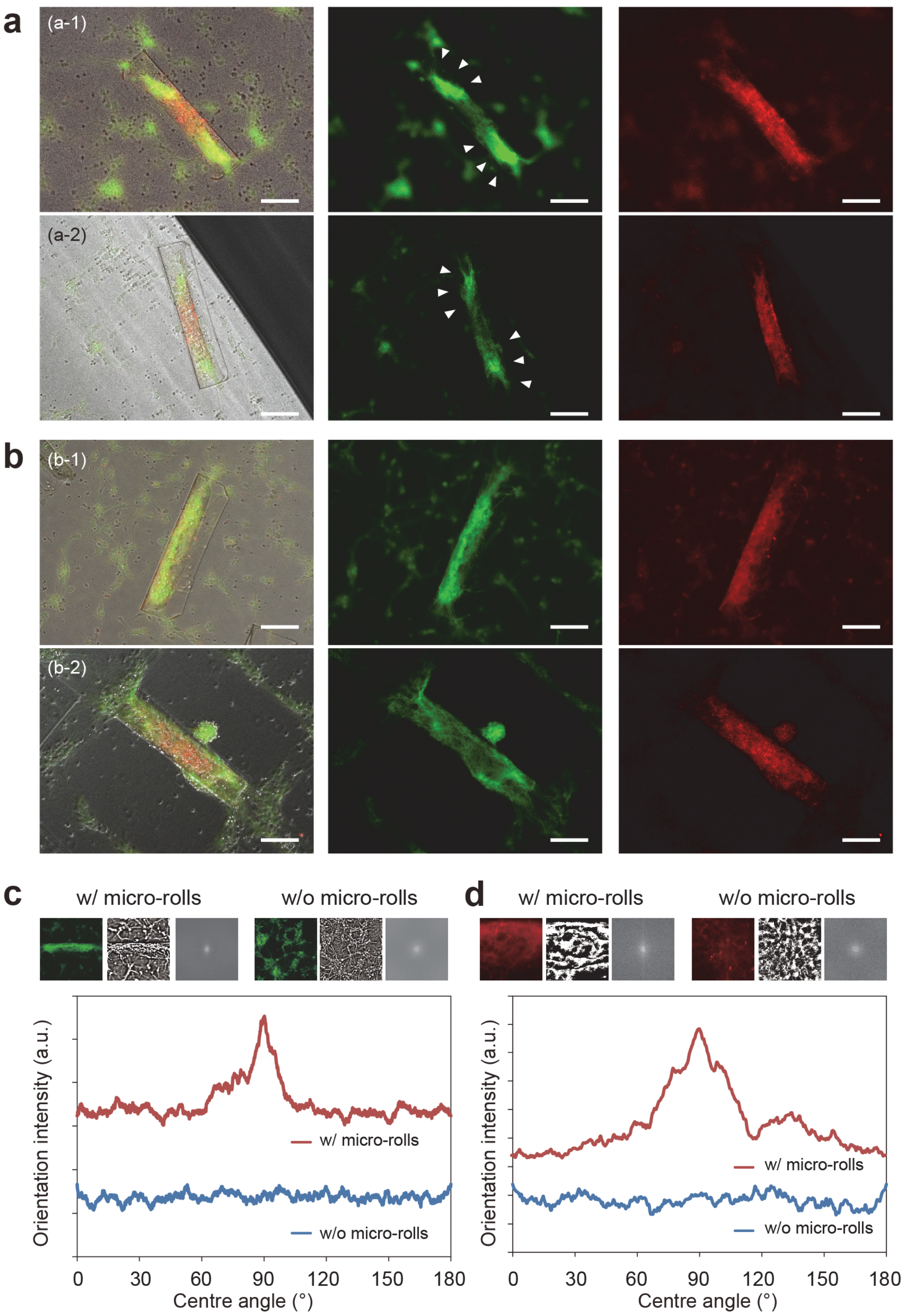

Supplementary Figure S9. Fluorescent observation of fluorescent-labelled hippocampal cells inside micro-rolls. **a-b**, Red (right) and green (centre)

fluorescence images and merged fluorescence and phase-contrast images (left) of immunostained hippocampal cell aggregations within micro-rolls. The somas and their neurites were visualised by using Alexa Fluor 488-conjugated Pan Neuronal Marker monoclonal antibody (centre) and Alexa Fluor 594-conjugated phalloidin (right). Since the location of spheroids of hippocampal cells was random, some spheroids were located in the edge of micro-rolls (a-1, a-2, centre), and the others were evenly distributed inside the micro-rolls (b-1, b-2, centre). **c-d**, The procedure for FFT analysis of immunostained hippocampal cells and their neurites that were evenly within the micro-rolls. The above sequential images are fluorescence, binarised, and FFT images. The graph below is corresponding summed pixel intensities from the FFT images along a straight line radiating at the angle for (c) neurites (1024 × 1024 pixels) and (d) actin fibres (256 × 256 pixels). Scale bars are 100 μm.

Supplementary Movie SM6. Raw colour movies and processed black-and-white movies of beating cardiomyocytes within the micro-rolls. Time-lapsed fluorescence images of Ca^{2+} oscillations in Fluo-4-labelled cardiomyocytes within the micro-rolls. The movies are played at normal speed.

Supplementary Movie SM7. Time-lapsed images of formation of cell aggregations and elongated neurons inside micro-rolls, confocal *z*-stack images of calcein-AM-labelled cells, and time-lapsed fluorescent images of Ca^{2+} oscillations in Fluo-4-labelled cells.

3. Supplementary references

1. Teshima, T., *et al.* High-resolution vertical observation of intracellular structure using magnetically responsive microplates. *Small* **12**, 3366-3373 (2016).
2. Teshima, T., *et al.* Mobile silk fibroin electrode for manipulation and electrical stimulation of adherent cells. *Adv. Funct. Mater.* **26**, 8185-8193 (2016).
3. Meng, E., Li P. Y. & Tai Y. C. Plasma removal of parylene-C. *J Micromech. Microeng.* **18**, 045004 (2008).
4. Lu, B., Zheng S., Quach B. Q. & Tai Y. C. A study of the autofluorescence of parylene materials for microTAS applications. *Lab Chip* **10**, 1826-1834 (2010).
5. 5. Li, Y., *et al.* Surface-enhanced molecular spectroscopy (SEMS) based on perfect-absorber metamaterials in the mid-infrared. *Sci. Rep.* **3**, 2865 (2013).
6. Behrens, S. H. & Grier D. G. The charge of glass and silica surfaces. *J. Chem. Phys.* **115**, 6716-6721 (2001).
7. Rizzi, F., Qualtieri A., Chambers L. D., Megill W. M. & De Vittorio M. Parylene conformal coating encapsulation as a method for advanced tuning of mechanical properties of an artificial hair cell. *Soft Matter* **9**, 2584-2588 (2013).
8. Xie, J., Shih J., Lin Q. A., Yang B. Z. & Tai Y. C. Surface micromachined electrostatically actuated micro peristaltic pump. *Lab Chip* **4**, 495-501 (2004).
9. Sim, W., Kim B., Choi B. & Park J. O. Theoretical and experimental studies on the parylene diaphragms for microdevices. *Microsyst. Technol.* **11**, 11-15 (2005).
10. White, R. D., *et al.* Rapid nano impact printing of silk biopolymer thin films. *J Micromech. Microeng.* **21**, 115014 (2011).
11. Bhattacharjee, M., *et al.* Oriented lamellar silk fibrous scaffolds to drive cartilage matrix orientation: Towards annulus fibrosus tissue engineering. *Acta Biomater.* **8**, 3313-3325 (2012).
12. Altman, G. H., *et al.* Silk matrix for tissue engineered anterior cruciate ligaments. *Biomaterials* **23**, 4131-4141 (2002).
13. Timoshenko, S. Analysis of bi-metal thermostats. *J. Opt. Soc. Am.* **11**, 233-255 (1925).
14. Alben, S., Balakrisnan B. & Smela E. Edge effects determine the direction of bilayer bending. *Nano Lett.* **11**, 2280-2285 (2011).
15. Arayanarakool, R., Meyer A. K., Helbig L., Sanchez S. & Schmidt O. G. Tailoring three-dimensional architectures by rolled-up nanotechnology for

mimicking microvasculatures. *Lab Chip* **15**, 2981-2989 (2015).

16. Randall, C. L., Kalinin Y. V., Jamal M., Manohar T. & Gracias D. H. Three-dimensional microwell arrays for cell culture. *Lab Chip* **11**, 127-131 (2011).

17. Ishihara, K., Fujiike A., Iwasaki Y., Kurita K. & Nakabayashi N. Synthesis of polymers having a phospholipid polar group connected to a poly(oxyethylene) chain and their protein adsorption-resistance properties. *J. Polym. Sci. Pol. Chem.* **34**, 199-205 (1996).

18. Sumino, Y., Magome N., Hamada T. & Yoshikawa K. Self-running droplet: emergence of regular motion from nonequilibrium noise. *Phys. Rev. Lett.* **94**, 068301 (2005)
